# Optimizing a Fed-Batch High-Density Fermentation Process for Medium Chain-Length Poly(3-Hydroxyalkanoates) in *Escherichia coli*

**DOI:** 10.3389/fbioe.2021.618259

**Published:** 2021-02-26

**Authors:** Ryan A. Scheel, Truong Ho, Yuki Kageyama, Jessica Masisak, Seamus McKenney, Benjamin R. Lundgren, Christopher T. Nomura

**Affiliations:** ^1^Department of Chemistry, State University of New York College of Environmental Science and Forestry, Syracuse, NY, United States; ^2^Division of Applied Chemistry, Department of Engineering, Hokkaido University, Sapporo, Japan; ^3^Department of Biological Sciences, College of Science, University of Idaho, Moscow, ID, United States

**Keywords:** polyhydroxyalkanoates, copolymers, functionalized monomers, fatty acids, recombinant bacteria, bioprocess

## Abstract

Production of medium chain-length poly(3-hydroxyalkanoates) [PHA] polymers with tightly defined compositions is an important area of research to expand the application and improve the properties of these promising biobased and biodegradable materials. PHA polymers with homopolymeric or defined compositions exhibit attractive material properties such as increased flexibility and elasticity relative to poly(3-hydroxybutyrate) [PHB]; however, these polymers are difficult to biosynthesize in native PHA-producing organisms, and there is a paucity of research toward developing high-density cultivation methods while retaining compositional control. In this study, we developed and optimized a fed-batch fermentation process in a stirred tank reactor, beginning with the biosynthesis of poly(3-hydroxydecanoate) [PHD] from decanoic acid by β-oxidation deficient recombinant *Escherichia coli* LSBJ using glucose as a co-substrate solely for growth. Bacteria were cultured in two stages, a biomass accumulation stage (37°C, pH 7.0) with glucose as the primary carbon source and a PHA biosynthesis stage (30°C, pH 8.0) with co-feeding of glucose and a fatty acid. Through iterative optimizations of semi-defined media composition and glucose feed rate, 6.0 g of decanoic acid was converted to PHD with an 87.5% molar yield (4.54 g L^–1^). Stepwise increases in the amount of decanoic acid fed during the fermentation correlated with an increase in PHD, resulting in a final decanoic acid feed of 25 g converted to PHD at a yield of 89.4% (20.1 g L^–1^, 0.42 g L^–1^ h^–1^), at which point foaming became uncontrollable. Hexanoic acid, octanoic acid, 10-undecenoic acid, and 10-bromodecanoic acid were all individually supplemented at 20 g each and successfully polymerized with yields ranging from 66.8 to 99.0% (9.24 to 18.2 g L^–1^). Using this bioreactor strategy, co-fatty acid feeds of octanoic acid/decanoic acid and octanoic acid/10-azidodecanoic acid (8:2 mol ratio each) resulted in the production of their respective copolymers at nearly the same ratio and at high yield, demonstrating that these methods can be used to control PHA copolymer composition.

## Introduction

High-density fermentation of *Escherichia coli* has been studied for the last 50 years in an effort to achieve maximal cell densities (∼200 g L^–1^ dry cell weight), frequently to attain high volumetric productivity (g L^–1^ h^–1^) of a heterologously expressed product (Shiloach and Fass, 2005). While this is often a product such as a protein or antibiotic, these techniques have also been employed to produce biopolymers such as poly(hydroxyalkanoates) [PHAs]. PHAs are a broad class of bacterially derived polyesters prized for their biodegradability and biocompatibility, which can be produced from renewable carbon sources such as sugars and lipids (Lu et al., 2009). There are two dominant types of PHA characterized by the number of carbons present in each monomer, short chain-length (SCL) PHA containing 3–5 carbons, and medium chain-length (MCL) PHA containing 6–14 carbons. The most commonly discussed PHAs in the literature are poly[(*R*)-3-hydroxybutyrate] (PHB) and SCL copolymers of PHB with 3-hydroxyvalerate, which exhibit stiff and brittle material properties with a high Young’s modulus (i.e., the measured stiffness of a material; Lu et al., 2009), while MCL PHAs and SCL-*co*-MCL copolymers are the subject of more recent studies due to their more viscoelastic properties (Rai et al., 2011; Tappel et al., 2012a).

Control over the monomeric composition of MCL PHAs can be achieved by culturing β-oxidation deficient (Δ*fadBJ*) *E. coli* LSBJ harboring the PHA biosynthesis genes *phaJ4* and *phaC1* (STQK) in the presence of fatty acid substrates as previously described by our laboratory (Tappel et al.,2012a,b). Controlling the monomer composition confers control over the material properties, which can be further customized by the incorporation of functionalized fatty acids that enable post-production chemical modifications (Levine et al., 2015; Pinto et al., 2016). One of the main limitations of this MCL PHA biosynthesis platform is the low polymer yields obtained; the first reported MCL PHA biosynthesis in *E. coli* LSBJ reported yields from shake flask cultivations of approximately 0.26–0.4 g L^–1^ (Tappel et al., 2012b), which were later improved slightly by the deletion of the *arcA* transcriptional regulator to 0.26–0.6 g L^–1^ (Scheel et al., 2016), and most recently improved to 5.44 g L^–1^ by utilizing glucose as a co-substrate, doubling the culture duration, and heterologously expressing an acyl-CoA synthetase from *Pseudomonas putida* (Mohd Fadzil et al., 2018).

Cultivating PHA-producing bacteria to a high-density in stirred tank reactors (STR, bioreactors) rather than shake flasks has been shown to greatly enhance volumetric productivity, and is a necessary step to scaling up production (Lee et al., 1999; Blunt et al., 2018; Koller, 2018). However, the most common products created using these high-density fermentation techniques are SCL PHAs and MCL ter- and quadripolymers with uncontrolled monomer composition, with only a few reports of successful MCL biosynthesis with controlled compositions. One notable exception is the biosynthesis of near-homopolymeric poly(3-hydroxydecanoate; PHD) reported by Gao et al. (2018), who obtained a yield of 11.8 g L^–1^ (0.41 g L^–1^ h^–1^) from β-oxidation deficient *P. putida* KT2440.

**FIGURE 1:**
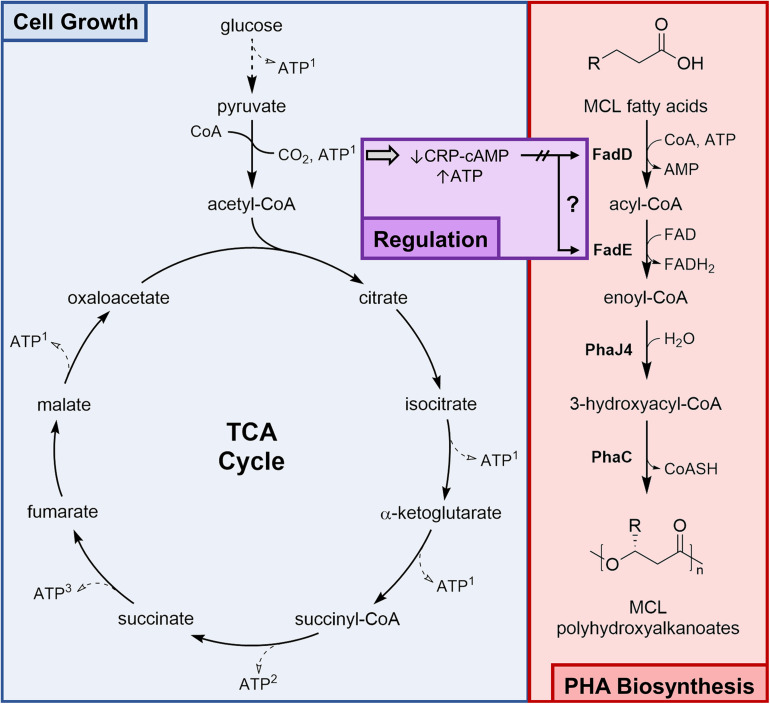
Simultaneous glucose consumption and PHA biosynthesis in *E. coli* LSBJ. Glucose is metabolized solely for cell growth, generating ATP and reducing equivalents *via* glycolysis and the tricarboxylic acid (TCA) cycle. Extracellular fatty acids (*R* = 1–9 carbon) cross the outer membrane by diffusion and are simultaneously transported and activated by the acyl-CoA synthetase FadD (Kameda and Nunn, 1981; Lepore et al., 2011). Acyl-CoAs are converted to enoyl-CoAs by the acyl-CoA dehydrogenase FadE and are unable to proceed by native β-oxidation due to the absence of FadB and FadJ (Tappel et al., 2012b). Recombinant enzymes PhaJ4 and PhaC1 (STQK), an *R*-specific enoyl-CoA hydratase and an engineered PHA synthase, respectively, are conferred by the pBBR-C1J4SII plasmid and complete the PHA biosynthesis pathway (Table 1). MCL PHA biosynthesis from fatty acids is largely uncoupled from cell growth; however, both *fadD* and *fadE* expression are activated by the binding of the cyclic AMP receptor protein – cyclic AMP (CRP-cAMP) complex (Feng and Cronan, 2012). A high ATP concentration inversely lowers cAMP concentration, reducing the amount of complexed CRP and potentially decreasing the expression of *fadD* and *fadE* and preventing PHA biosynthesis. ATP shown in this figure is a product of the intermediates NADH^1^, GTP^2^, and FADH_2_^3^ (with the exception of some ATP generated by glycolytic enzymes).

The goal of this study was to optimize a fed-batch fermentation process for the high-density cultivation of recombinant *E. coli* LSBJ to produce MCL PHA polymers with controlled composition from fatty acid substrates. PHA biosynthesis from fatty acids is mostly decoupled from primary metabolism; exogenous fatty acids are imported and converted into 3-hydroxyacyl-CoA *via* three steps of a modified β-oxidation pathway and are polymerized by the PHA synthase PhaC1 (STQK), while glucose catabolism drives cell growth and provides ATP and reducing equivalents (Figure 1). Catabolite repression induced by the presence of glucose is one regulatory network expected to bridge the gap between primary metabolism and β-oxidation. Fatty acid metabolism is tightly regulated in *E. coli* and is subject to catabolite repression through the activity of the cyclic AMP receptor protein – cyclic AMP complex (CRP-cAMP), a global transcriptional regulator that activates transcription of *fad* genes in response to elevated cAMP levels in the absence of glucose; this activation is lost when glucose is present in sufficient concentrations (Iram and Cronan, 2006; Deutscher, 2008; Fic et al., 2009; Feng and Cronan, 2012).

The work presented in this study demonstrates the first successful biosynthesis of MCL PHA homopolymers and copolymers with defined monomer composition in *E. coli* LSBJ *via* high-density fermentation, and a subsequent process optimization to increase PHA yields and volumetric productivity. PHD was ultimately chosen as a model MCL homopolymer for the optimization experiments due to its increased crystallinity at high monomer ratios, which enhances thermomechanical properties typically lacking in MCL PHAs (e.g., low tensile strength, low melting temperature), and because PHD homopolymers have been previously produced by *E. coli* LSBJ in batch shake flask fermentations at high yields (Liu et al., 2011; Tappel et al., 2012b).

## Materials and Methods

### Media and Inoculum Preparation

A complete list of strains, plasmids, and primers used for this study can be found in Table 1. Unless otherwise specified, all strains were maintained in LB-Lennox (LB; composition per liter: 10 g tryptone, 5 g yeast extract, and 5 g NaCl, pH 7.0) purchased from Difco, and the antibiotics kanamycin (50 mg L^–1^) and ampicillin (100 mg L^–1^) were added to media as necessary. When necessary, Bacto agar (Difco) was added to media at a concentration of 15 g L^–1^ to make plates.

**TABLE 1:** Strains, plasmids, and primers.

*Escherichia coli*	Relevant characteristics	Source or reference
LSBJ	*ΔfadB, ΔfadJ, atoC512* (Const), *fadR601*	Tappel et al., 2012b
RSC02	Δ*arcA* LSBJ	Scheel et al., 2016
LSBJ Δ*crp*	Δcrp LSBJ	This study
LSBJ CRP*	crp*(I112L, T127I, A144T) LSBJ	This study
SM02	Δ*dld* LSBJ CRP*	This study
SM04	*Δdld, ΔarcA* LSBJ CRP*	This study
SM06	*Δdld, ΔarcA, Δaas* LSBJ CRP*	This study
*Plasmids*		
pKD46	λ Red recombinase expression plasmid; expresses *exo*, β, and γ genes from λ phage; P_*araB*_ promoter; *araC*; Amp^*R*^; temperature sensitive replicon	Datsenko and Wanner, 2000
pKD13	Neomycin phosphotransferase flanked by FLP recombinase recognition targets, Amp^*R*^, Km^*R*^	Datsenko and Wanner, 2000
pCP20	FLP recombinase expression plasmid, Amp^*R*^, temperature sensitive replicon	Datsenko and Wanner, 2000
pBBR-C1J4SII	pBBR1MCS-2 derivative, *phaJ4, phaC1* (STQK)	Tappel et al., 2012b
pZS411	pTrc99a derivative, *pct* (*Megasphaera elsdenii*)	This study
pRAS02	pJet1.2 derivative, native *crp* (*E. coli* MG1655)	This study
pRAS05	pJet1.2 derivative, engineered crp*	This study
pTrcDAEB	pTrc99a derivative, *atoDAEB* operon (*E. coli* MG1655)	This study
	
*Primers*^*a,b,c*^	Sequence (5′ to 3′)	
pKD13.F.*arcA*	ATGCAGACCCCGCACATTCTTATCGTTGAAGACGAGTTGGTAACACGCAAGTGTAGGCTGGAGCTGCTTC	
pKD13.R.*arcA*	TTAATCTTCCAGATCACCGCAGAAGCGATAACCTTCACCGTGAATGGTGGATTCCGTGGATCCGTCGACC	
pKD13.F.*aas*	AATCTCCCTCCATTCGCTTTTACTGAATCAGAGCAAAGGGAGTTGGAATGTGTAGGCTGGAGCTGCTTC	
pKD13.R.*aas*	ACCACAACGAAGTGTTAGTGTGCACTGACTCACTCATCGTGTTGTTCCGCATTCCGTGGATCCGTCGACC	
pKD13.F.*crp*	ATGGTGCTTGGCAAACCGCAAACAGACCCGACTCTCGAATGGTTCTTGTCGTGTAGGCTGGAGCTGCTTC	
pKD13.R.*crp*	TTAACGAGTGCCGTAAACGACGATGGTTTTACCGTGTGCGGAGATCAGGTATTCCGTGGATCCGTCGACC	
*crpM1*.F/R	AAACCCGGACcttCTGATGCGTT/ACCTGAATCAATTGGCGAAATTTTTTG	
*crpM2*.F/R	TCTGCAAGTCattTCAGAGAAAGTGGG/CGACGCGCCATCTGTGCA	
*crpM3*.F/R	GGGCCGCATTacaCAGACTCTGC/GTCACGTCGAGGAACGCC	
native.*crp*.F/R	ATGGTGCTTGGCAAACCGCA/TAAACGAGTGCCGTAAACGAC	
*arcA*.check.F/R	GTTAATTTGCAGCATGCATCAGG/GACGATGAGTTACGTATCTGG	
*aas*.check.F/R	AAATTGTTGCGAACCTTTGG/GCAATAACGTACGGGCAAAT	
*crp*.check.F/R	ATCCAGAGACAGCGGCGTTA/AATGGCGCGCTACCAGGTAA	

*^*a*^Underlined sequences are homologous to the gene to be deleted. ^*b*^Forward and reverse primers are denoted with an F or R, respectively. ^*c*^Lowercase base-pairs indicate codons subjected to site-directed mutagenesis.*

The fatty acids hexanoic acid (Sigma Aldrich), sodium octanoate (Sigma Aldrich), decanoic acid (Alfa Aesar), and 10-undecenoic acid (Sigma Aldrich). 10-bromodecanoic acid (A.K. Scientific), and 10-azidodecanoate (synthesized as described below) were supplemented as substrates for polymer production as noted. Fatty acid feed solutions for bioreactor fermentations were brought to pH 8.0 by the addition of NaOH (10 M) to form the respective conjugate base and either autoclaved (saturated fatty acids) or filter-sterilized (functionalized fatty acids) prior to addition to the bioreactor.

### *E. coli* Engineering and Plasmid Construction

The *dld*, *arcA*, and *aas* genes were deleted using homologous recombination *via* the λ Red recombinase protocol described by Datsenko and Wanner (2000). Knockout cassettes were generated by PCR (PrimeSTAR HS polymerase, Takara) using gene-specific primers to amplify the kanamycin resistance marker from pKD13, gel-purified, and concentrated by ethanol precipitation. Knockout cassettes were introduced to parental strains harboring pKD46 by electroporation (1500V, 5 m; BTX ECM 399), recovered in 1 mL LB at 30°C and 250 rpm for 1 h, and plated on LB agar supplemented with kanamycin at 37°C for 24 h. For the *dld* knockout, colonies were patched onto minimal media agar plates (22 mM KH_2_PO_4_, 42 mM Na_2_HPO_4_, 8.6 mM NaCl, 2.0 mM MgSO_4_, 18.9 mM NH_4_Cl, 15 g L^–1^ agar, pH 7.0) that were supplemented with 25 mM D-lactate. Colonies that did not grow on the D-lactate minimal agar plates were considered putative *dld*::Km^*R*^ mutants. Successful gene replacement for the *arcA*::Km^*R*^ and *aas*::Km^*R*^ mutants was confirmed by loci screening using PCR. The temperature-sensitive pKD46 plasmid was removed by growth for 12 h at 42°C. Antibiotic markers were removed by expression of FLP recombinase from the pCP20 plasmid, which was confirmed by the loss of kanamycin resistance and loci screening by PCR. The temperature-sensitive pCP20 plasmid was removed by growth for 12 h at 42°C. Successful mutants were prepared as 30% glycerol stocks and stored at –80°C.

The *crp*^∗^ mutation, a group of mutations that exhibit reduced dependence on cAMP for DNA binding and subsequent transcriptional regulation (Guidi-Rontani et al., 1981; Khankal et al., 2009), was introduced to *E. coli* LSBJ by first deleting the native *crp* gene and then replacing it with the mutated *crp*^∗^ gene. Native *crp* was removed following the same λ Red recombinase protocol described above. The *crp*^∗^ gene was generated from the native *E. coli* MG1655 *crp* gene using the Q5^®^ Site-Directed Mutagenesis Kit (New England Biolabs) following manufacturers recommendations. In brief, native *crp* was PCR amplified from MG1655 genomic DNA using the native.*crp.*F/native.*crp.*R primers and blunt ligated into pJet1.2/blunt to generate pRAS02. This plasmid was subjected to three successive rounds of site-directed mutagenesis using the *crp*M1, *crp*M2, and *crp*M3 primer sets to make the following substitutions, respectively: I112L, T127I, and A144T. The final *crp*^∗^ gene harboring all three mutations, designated pRAS05, was verified by Sanger sequencing (GENEWIZ, NJ, United States).

The *crp*^∗^ gene was PCR amplified from pRAS05 using the native.*crp.*F/native.*crp.*R primers. The PCR mixture was treated with Dpn I and the desired ∼700 bp *crp*^∗^ product was gel purified. The purified *crp*^∗^ was concentrated *via* ethanol precipitation and subsequently electroporated into LSBJ Δ*crp* cells harboring the λ Red plasmid pKD46. Cells were recovered in 1 mL of LB at 30°C and 250 rpm for 1 h, and 0.5 mL of recovery culture was harvested by centrifugation and suspended in 0.2 mL of sterile water. Cell suspension (0.1 mL) was plated onto minimal medium agar consisting of 22 mM KH_2_PO_4_, 42 mM Na_2_HPO_4_, 8.6 mM NaCl, 2.0 mM MgSO_4_, 20 mM pyruvate (carbon source), and 20 mM L-tryptophan (nitrogen source). This selection method was chosen because *crp* is required for growth on L-tryptophan as a nitrogen source in the presence of pyruvate as a carbon source (Isaacs et al., 1994). Plates were incubated at 37°C for 48 h, and colonies visible after 48 h were patched onto LB agar plates and subsequently screened *via* Sanger sequencing to verify the presence of the *crp*^∗^ gene. The LSBJ CRP^∗^ mutant was prepared as a 30% glycerol stock and stored at –80°C.

The pZS411 plasmid, harboring the *pct* gene necessary for lactyl-CoA formation from lactate, was constructed by excising the *pct* gene from the pTV118NpctC1AB(STQK) plasmid (Taguchi et al., 2008) using 5′ and 3′ *Eco*RI sites and ligating into pTrc99a following standard cloning procedures (Sambrook and Russell, 2001). The pTrcDAEB plasmid was constructed by amplifying the *atoDAEB* operon using the *atoDAEB.*F/R primer set and ligating this fragment into the blunt cloning vector pJet1.2/blunt following manufacturer’s specifications. The operon was then excised with 5′ *Sac*I and 3′ *Kpn*I, purified by gel electrophoresis, and ligated into pTrc99a.

### PHA Production in Shake Flasks

Poly(3-hydroxyalkanoates) production in shake flasks was performed as previously described (Scheel et al., 2016). In brief, chemically competent *E. coli* LSBJ was transformed with MCL PHA biosynthesis plasmid pBBR-C1J4SII by heat-shock following standard procedures (Sambrook and Russell, 2001). Transformants were grown on LB-agar plates, and single colonies were used to inoculate three separate 2.5 mL LB seed cultures. Seed cultures were grown for 16 h at 37°C and 200 rpm rotary shaking, and 500 μL were used to inoculate 100 mL of growth media (LB, 4.0 g L^–1^ Brij-35, 10 mM decanoic acid, 8 mM sodium phosphate dodecahydrate, kanamycin, pH 7.0) in 500-mL baffled shake flasks. Cultures were grown for 12, 24, 36, or 48 h at 30°C and 220 rpm, then harvested by centrifugation as previously described (Tappel et al., 2012b). Before harvesting, the pH of each culture was measured (Accumet^®^ pH meter, Fisher Scientific) immediately after removal from the incubator.

### Low-Density Fed-Batch Production

**FIGURE 2:**
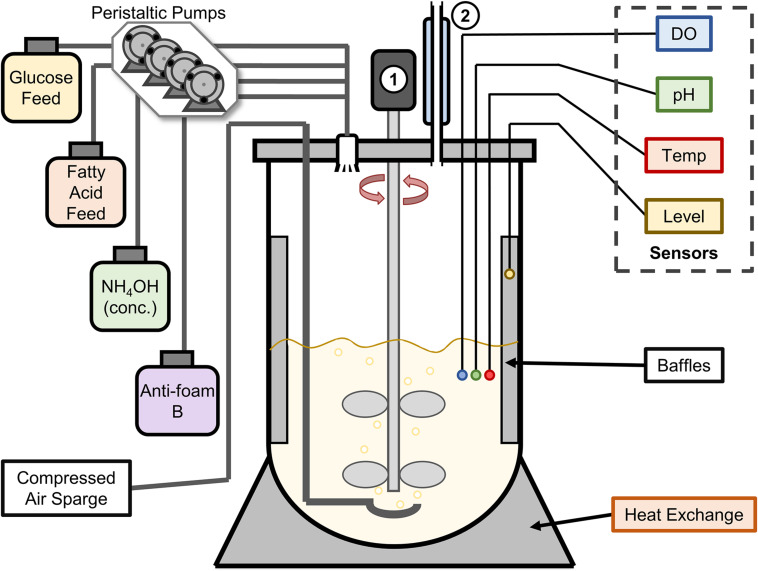
Schematic of the New Brunswick BioFlo 310 2L bioreactor vessel used for fermentation experiments. Four sensors were installed to measure dissolved oxygen (DO), pH, temperature (Temp), and the culture level (for foam detection). Glucose and fatty acid feeds were added at a set rate by peristaltic pumps, while concentrated ammonium hydroxide and 10% Antifoam B in water were added automatically by peristaltic pumps controlled by their respective sensors (pH and Level). The numbers **1** and **2** denote the motor/impeller system and a water-cooled condenser, respectively.

A summary of all bioreactor trials outlining the *E. coli* strains, plasmids, and conditions can be found in Table 2. All fed-batch fermentations were performed in a 2-L vessel with a BioFlo 310 benchtop bioreactor system (New Brunswick Scientific) using conditions and media adapted from Pfeifer et al. (2002). A schematic of this bioreactor assembly is shown in Figure 2. For initial experiments, the assembled bioreactor containing 750 mL of defined medium [F1 Salts; 0.4 g L^–1^ (NH_4_)_2_SO_4_, 1.5 g L^–1^ KH_2_PO_4_, and 4.35 g L^–1^ K_2_HPO_4_, pH 7.0] was autoclaved at 121°C for 35 min. Prior to inoculation, 7.5 g glucose, 1.5 g yeast extract, 0.12 g MgSO_4_, 1 mL of 0.05 g L^–1^ kanamycin sulfate, and 0.75 mL of trace metal solution were added aseptically. Trace metal solution contained 5 g L^–1^ NaCl, 1 g L^–1^ ZnSO_4_ ⋅ 7H_2_O, 4 g L^–1^ MnCl_2_ ⋅ 4H_2_O, 4.75 g L^–1^ FeCl_3_, 0.4 g L^–1^ CuSO_4_ ⋅ 5H_2_O, 0.58 g L^–1^ H_3_BO_3_, 0.5 g L^–1^ NaMoO_4_ ⋅ 2H_2_O, and 8 mL L^–1^ concentrated H_2_SO_4_. The bioreactor was inoculated with 40 mL of bacterial seed culture, which was grown in LB for 16 h at 37°C and 200 rpm rotary shaking, pelleted at 3,716 × *g* for 10 min, and resuspended in 2 mL PBS buffer before sterile transfer to the bioreactor vessel.

**TABLE 2:** Summary of bioreactor conditions.

Trial^1^	Strain	Plasmid	Glucose feed rate (mL h^–1^)	Fatty acid feed^3^; total mass (g)	Fatty acid feed rate (mL h^–1^)	pH	Final culture volume (L)
1	SM02	pBBRC1J4SII/pZS411	14.5	C8; 0.8	Bulk	7	
2	SM04	pBBRC1J4SII	14.5	C8; 6	10	7	
3	SM06	pBBRC1J4SII	14.5/9.7	C8; 1.0	Bulk	7	
4	SM06	pBBRC1J4SII	14.5/9.7	C8; 1.5	Bulk	7	
5	SM06	pBBRC1J4SII	14.5/9.7	C8; 6	8.3	7	
6	SM06	pBBRC1J4SII	14.5/9.7	C8; 6	5.5	7	
7^4^	SM06	pBRL690	14.5/9.7	C8; 6	5.5	7	
8^4^	LSBJ	pBBRC1J4SII/pTrcDAEB	14.5	C10; 6	5.5	7	
SF-1	LSBJ	pBBRC1J4SII	NA	C10; 0.17	Bulk	7.6	
SF-2	LSBJ	pBBRC1J4SII	NA	C10; 0.17	Bulk	8.5	
SF-3	LSBJ	pBBRC1J4SII	NA	C10; 0.17	Bulk	8.7	
SF-4	LSBJ	pBBRC1J4SII	NA	C10; 0.17	Bulk	8.8	
9	LSBJ	pBBRC1J4SII	14.5	C10; 6	5.5	7/8	0.94 ± 0.01
10	RSC02	pBBRC1J4SII	14.5	C10; 6	5.5	7/8	0.85 ± 0.004
11	LSBJ CRP*	pBBRC1J4SII	14.5	C10; 6	5.5	7/8	1.12 ± 0.03
12	LSBJ	pBBRC1J4SII	14.5/9.7	C10; 6	5.5	7/8	1.12 ± 0.10
13	LSBJ	pBBRC1J4SII	14.5/9.7	C10; 6	5.5	7/8	1.14 ± 0.02
14	LSBJ	pBBRC1J4SII	14.5/9.7	C10; 12	5.5	7/8	1.19
15^2^	LSBJ	pBBRC1J4SII	14.5/9.7	C10; 12	5.5	7/8	1.05 ± 0.17
16	LSBJ	pBBRC1J4SII	14.5/9.7	C10; 15	5.5	7/8	1.00 ± 0.0
17	LSBJ	pBBRC1J4SII	12.9/8.7	C10; 20	6.5	7/8	0.98 ± 0.06
18	LSBJ	pBBRC1J4SII	11.4/7.7	C10; 25	7.5	7/8	1.10
19	LSBJ	pBBRC1J4SII	12.9/8.7	C6; 20	6.5	7/8	1.07
20	LSBJ	pBBRC1J4SII	12.9/8.7	C8; 20	6.5	7/8	1.04
21	LSBJ	pBBRC1J4SII	12.9/8.7	C11Δ10; 20	6.5	7/8	1.43
22	LSBJ	pBBRC1J4SII	12.9/8.7	C10Br; 20	6.5	7/8	1.26
23	LSBJ	pBBRC1J4SII	12.9/8.7	C8/C10; 15.4/4	6.5	7/8	1.00
24	LSBJ	pBBRC1J4SII	12.9/8.7	C8/C10N_3_; 15.4/4.95	6.5	7/8	0.95

*^1^Trials 1–8 are preliminary experiments which did not result in polymer production, and no final culture volume was recorded for these trials. Trials 1–12 were performed following low-density fermentation methods, while trials 13–24 were performed following high-density fermentation methods. Trials 9–17 were performed in duplicate as two consecutive bioreactor fermentations, with the exception of trial 14, and culture volumes are reported as the average (L) ± the standard deviation for the two fermentations. Trials SF-1 through SF-4 describe the four timepoints of the Shake Flask experiment (12, 24, 36, and 48 h, respectively) shown in Figure 4B, and were added for the sake of comparison.*

*^2^Yeast extract in culture medium was doubled to 3.0 *g* total for trial 15, and this amount was maintained for each subsequent trial.*

*^3^Fatty acids are abbreviated as follows: octanoic acid (C8), decanoic acid (C10), hexanoic acid (C6), 10-undecenoic acid (C11Δ10), 10-bromodecanoic acid (C10Br), and 10-azidodecanoic acid (C10N_3_). Total feed volume was 150 mL for Trials 2–16, increased to 200 mL for Trial 17 and 250 mL for Trial 18 in order to dissolve the greater mass of decanoic acid. Trials 19–24 were reduced back to 200 mL.*

*^4^Isopropyl β-D-1-thiogalactopyranoside (IPTG) added to Trials 7 and 8 at a final concentration of 0.5 mM to induce pBRL690 and pTrcDAEB expression, respectively.*

Fermentation was carried out in two stages, a biomass accumulation stage (Stage 1) and a polymer biosynthesis stage (Stage 2). The first stage of fermentation was performed at 37°C with an initial agitation rate of 200 rpm and an aeration rate of 5.0 L min^–1^, and pH was maintained at 7.0 by automated addition of concentrated NH_4_OH (17 M) as monitored by an internal pH meter. Dissolved oxygen was maintained at 50% air saturation controlled by agitation and air (compressed atmospheric) sparging cascades (200 to 950 rpm, then 5.0 to 20.0 L min^–1^ air flow). Foaming was controlled by automated addition of Antifoam B (J.T. Baker, 10% v/v unless otherwise noted). Once the initial glucose was consumed (as evidenced by a sudden increase in dissolved oxygen), a glucose feed solution (500 mL, filter-sterilized) consisting of 430 g L^–1^ glucose, 3.9 g L^–1^ MgSO_4_, 110 g L^–1^ (NH_4_)_2_SO_4_, and 50 mg L^–1^ kanamycin was supplied at a rate of 14.5 mL h^–1^ with a separate peristaltic pump. OD_600_ measurements were made on 1 mL culture aliquots each hour using a Genesys 10S UV-Vis spectrophotometer (Thermo Scientific).

After approximately 12 h of growth in Stage 1 (OD_600_ > 30), Stage 2 was initiated by decreasing the fermentation temperature to 30°C, increasing the pH setpoint to 8.0 (with the exception of preliminary experiments, Table 2), decreasing the dissolved oxygen setpoint to 30% air saturation, and beginning the addition of a fatty acid feed solution supplied at a rate of 5.5 mL h^–1^ (except where otherwise noted for preliminary experiments) with another peristaltic pump. For Trial 1, D-lactate (11 g) was added alongside octanoic acid as a bulk supplement. Glucose feed addition during the second stage was either maintained at 14.5 mL h^–1^ or reduced to 9.7 mL h^–1^ where noted. Culture samples (10 mL) were removed at 24 and 48 h and stored at –80°C for later quantitative PHA analysis. The remaining culture at 48 h was harvested as described below.

### High-Density Fed-Batch Production

High-density fermentations were carried out in the same 2-L vessel, using media adapted from Lau et al. (2004). The F1 Salts medium was replaced with 750 mL of defined medium consisting of 0.4 g L^–1^ (NH_4_)_2_SO_4_, 4.16 g L^–1^ KH_2_PO_4_, and 11.95 g L^–1^ K_2_HPO_4_. Prior to inoculation, 7.5 g glucose, 1.5 g yeast extract, 0.12 g MgSO_4_, 1 mL of 0.05 g L^–1^ kanamycin sulfate, and 2.25 mL of trace metal solution were added aseptically. The amount of yeast extract was increased to 3.0 g for trial 15 and for each subsequent trial (Table 2). The amount of (NH_4_)_2_SO_4_ in the glucose feed was reduced to 6 g L^–1^, and 15 mL of trace metal solution was added to the feed as well. The transition to Phase 2 was made consistently at 12 h rather than in response to the OD_600_. All other bioreactor conditions were the same as for the low-density production process.

Fatty acid feed solutions initially contained 40 g L^–1^ decanoic acid (6 g in 150 mL H_2_O, pH 8.0), and were raised in subsequent trials as shown in Table 2. To maintain a constant volume of the combined glucose and fatty acid feeds, as fatty acid feed volumes increased (due to a maximum decanoic acid solubility of 100 g L^–1^ at pH 8.0) the glucose feed volume was decreased by the same magnitude while holding the mass of feed components constant. To account for this change in glucose feed concentration, the addition rate was reduced proportionally so that the g glucose h^–1^ was kept constant. Similarly, fatty acid feed rates were increased so that the amount of fatty acid added per hour increased proportionally and to ensure that the entirety of the fatty acid solution was added to the culture prior to the end of fermentation at 48 h. Antifoam concentration was increased to 20% for Trials 18 and 24 to combat excessive foaming.

### PHA Quantification

Shake flask cultures were harvested as previously described (Scheel et al., 2019). In brief, cells were collected by centrifugation at 3716 × *g* for 15 min, washed once with 45 mL of 35% ethanol and once with 45 mL of Nanopure filtered water, frozen at –80°C, and dried *via* lyophilization.

The 10 mL culture samples from bioreactor fermentations were thawed and pelleted by centrifugation at 7,000 × *g* for 15 min. The supernatant was reserved and frozen at –80°C for lyophilization and later analysis. Cell pellets were washed once with 40% ethanol (40 mL) and once with Nanopure filtered water (40 mL), pelleting with the same centrifugation conditions after each wash. Pellets were then resuspended in 5 mL Nanopure filtered water, frozen at –80°C, and dried *via* lyophilization.

The PHA yields and repeating unit compositions were analyzed by GC as previously described (Scheel et al., 2019). In brief, lyophilized cells (10–15 mg) were suspended in 2 mL of a 15% (v/v) sulfuric acid solution in methanol and 2 mL of chloroform and heated at 100°C for 140 min in a 10 mL pressure vial (Kimax). The samples were cooled to room temperature, and 1 mL of Nanopure filtered water and 500 μL of methyl octanoate standard (0.25% v/v) in chloroform were added and mixed by vortex. Aqueous and organic layers were separated by centrifugation for 5 min at 700 rpm (Marathon 6K, Fisher Scientific). The organic layer was passed through a 0.2 μm polytetrafluoroethylene (PTFE) filter using a vacuum manifold (Millex Samplicity^®^) into 2 mL GC vials. Samples were injected and separated using a GC 2010 Gas Chromatograph (Shimadzu) with an AOC-20i autoinjector and a flame ionization detector. Shimadzu’s GCSolution software was used to analyze the data based on 3-hydroxyacyl methyl ester standard curves (except for 3-hydroxy-10-azidodecanoate monomers, which is described below). Residual fatty acids from the culture supernatant after 48 h were also analyzed by GC from 10 to 15 mg samples of lyophilized supernatant following the same protocol.

### 10-Azidodecanoic Acid Synthesis

**FIGURE 3:**
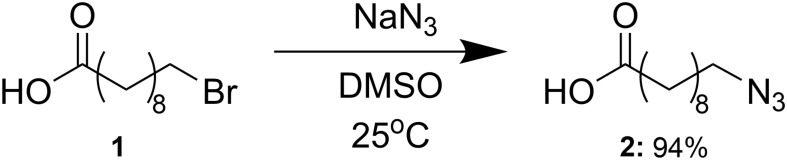
Synthesis of 10-azidodecanoic acid. Abbreviations: NaN_3_, sodium azide; DMSO, dimethylsulfoxide.

The 10-azidodecanoic acid substrate was synthesized following previously described methods (Riva et al., 2014), and is summarized in Figure 3. Sodium azide (15.01 g, 231 mmol) was added to a solution of 10-bromodecanoic acid (**1**, 40.00 g, 159 mmol) in dimethyl sulfoxide (1000 mL). The mixture was stirred at room temperature for 24 h and then water (2000 mL) was added. The aqueous solution was extracted with ethyl acetate (4 × 700 mL) and then washed with half-saturated brine (6 × 500 mL) and brine (2 × 400 mL) and dried over sodium sulfate. The sodium sulfate was filtered off and ethyl acetate was removed *in vacuo* to yield the azide (**2**, 32.84 g, 94%). Synthesis and purity were confirmed by ^1^H-NMR spectroscopy based on assigned chemical shifts supported by the literature (Supplementary Figure 1).

**FIGURE 4:**
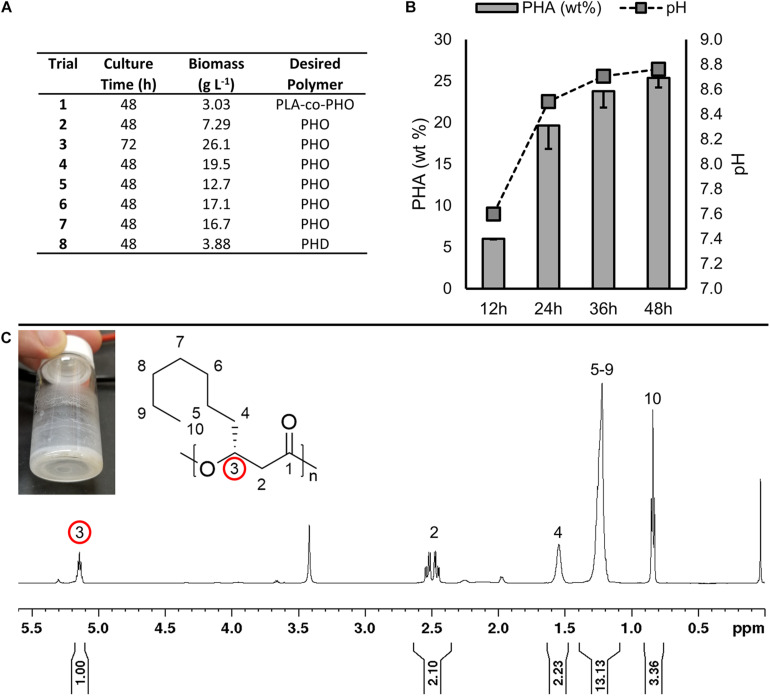
**(A)** Results of preliminary fermentations that did not result in polymer accumulation. **(B)** Culture pH and PHA accumulation in LSBJ harboring pBBRC1J4SII over 48 h growth in shake flasks, sampled every 12 h (Trials SF–1 through SF–4, respectively, Table 2). PHA was measured as a percent of dry cell weight. Data shown is the average of triplicate cultures; errors bars represent minus 1 standard deviation. **(C)** Photograph and ^1^H NMR (600 MHz) spectrum of PHD extracted from LSBJ-pBBRC1J4SII bioreactor fermentation (Trial 9). Diagnostic chemical shift for PHA at δ 5.17–5.13 ppm (pentet, 1H); 2.59–2.45 (multiplet, 2H), 1.61–1.47 (multiplet, 2H), 1.33–1.18 (multiplet, 10H), and 0.8–0.9 (triplet, 3H). Integration indicative of residual fatty acid.

### PHA Purification and Molecular Weight Analysis

Bioreactor cultures were harvested after 48 h by dividing the volume equally among 800-mL centrifuge buckets and centrifuging at 3,716 × *g* for 45 min. Cell pellets were washed once with 40% ethanol (approximately 400 mL) and once with Nanopure filtered water (400 mL), pelleting with the same centrifugation conditions after each wash. Final cell pellets were transferred to a tared 250-mL centrifuge tube, frozen at –80°C, and dried *via* lyophilization.

Poly(3-hydroxyalkanoates) was isolated from dried cells by Soxhlet extraction. Approximately 5–10 g of cells were transferred to 80 × 25 mm cellulose extraction thimbles (Whatman) and refluxed with 150 mL chloroform for 6 h. Crude polymer was concentrated by rotary evaporation and purified by dropwise addition to stirring methanol (180 mL, 4°C) without exceeding a 1:10 ratio of chloroform to methanol. The methanol solution was centrifuged at 7,000 × *g* and 4°C for 20 min to collect any suspended polymer. This methanol purification process was repeated twice, and purified PHA was dissolved in chloroform and passed through a syringe filter (0.45 μm PTFE) before drying *in vacuo*.

The weight average (M_*w*_) and number average (M_*n*_) molecular weights for each sample were determined by gel permeation chromatography (GPC) as previously described (Scheel et al., 2016). In brief, PHA solutions of approximately 5.0 g L^–1^ were prepared by dissolution in chloroform and passed through a syringe filter (0.45 μm PTFE). Samples were injected (50 μL) into a Shimadzu LC-20AD liquid chromatograph equipped with a Shimadzu SIL-20A autosampler, a Shimadzu CTO-20A column oven, and a Shimadzu RID-10A refractive index detector. Samples were passed through an 8 × 50 mm styrenedivinylbenzene (SDV) guard column (5 μm particles; Polymer Standards Service) and an 8 × 300 mm SDV analytical column (5 μm particles; mixed bed porosity; max molecular weight 1E6 Da; Polymer Standards Service product sda0830051lim). The column oven was maintained at 40°C with a 1 mL min^–1^ mobile phase of chloroform. Molecular weight standards of polystyrene with a narrow polydispersity index were used for calibration. LCsolution software (Shimadzu) was used for data analysis.

The presence of functional groups on applicable PHAs was verified by ^1^H-NMR spectroscopy using either a Bruker AVANCE III 600 MHz or Bruker AVANCE III HD 800 MHz instrument as noted. ^1^H-NMR was also used to confirm PHA production from the first successful bioreactor fermentation (Trial 9, Table 2). Spectra were processed with Bruker TopSpin v3.5pI2 and are available in the Supplementary Material.

### Calculations

Poly(3-hydroxyalkanoates) yields (g L^–1^) were calculated from the weight percent obtained using GC calibration curves as described above and the dried cell weight of 10.0 mL samples, dividing by the sample volume:

$$\mathrm{PHA}\,\mathrm{yield}\,\left(g\,L^{-1}\right)=\left(\frac{\text{Weight}\%\times \mathrm{Dried}\,\mathrm{Cell}\,\mathrm{Weight}\,\left(\text{g}\right)}{0.010\,L}\right)$$

The total PHA accumulated for each bioreactor fermentation was calculated by multiplying the PHA yield by the final culture volume from the bioreactor:

$$\mathrm{Total}\,\mathrm{PHA}\,\left(\text{g}\right)=\mathrm{PHA}\,\mathrm{yield}\,\left(\text{g}\,{\text{L}}^{-1}\right)\times \mathrm{Final}\,\mathrm{volume}\,\left(\text{L}\right)$$

Residual fatty acids remaining in the supernatant were calculated in the same manner as for PHA. The yield (g L^–1^) was first calculated from the weight percent obtained using GC calibration curves of pure fatty acid methyl esters and the dry weight of 10.0 mL lyophilized cell-free supernatant samples. This yield was then multiplied by the final culture volume (L) from the bioreactor to determine the total residual fatty acid:

$$\mathrm{Residual}\,\mathrm{fatty}\,\mathrm{acid}\,\left(\text{g}\right)=\left(\frac{\text{Weight}\%\times \mathrm{Dried}\,\mathrm{Supernatant}\,\left(g\right)}{0.010\,L}\right)\times \mathrm{Final}\,\mathrm{volume}\,\left(\text{L}\right)$$

Maximum theoretical yields of PHA were calculated from the total mass of fatty acid substrate available, multiplied by the molecular weight ratio of diradical monomeric equivalents to fatty acid precursor, assuming complete 1:1 conversion:

$$\text{Theoretical}\,\left(\text{g}\right)=\mathrm{Fatty}\,\mathrm{acid}\,\mathrm{substrate}\,\left(\text{g}\right)\times \left(\frac{M\,W_{\mathrm{diradical}\,\mathrm{monomer}}\,\left(g\,{\mathrm{mol}}^{-1}\right)}{M\,W_{\mathrm{fatty}\,\mathrm{acid}}\,\left(g\,{\mathrm{mol}}^{-1}\right)}\right)$$

The mole fraction (χ) of 3-hydroxy-10-azidodecanoate (DN_3_) monomers in the PHODN_3_ copolymer was calculated *via*^1^H-NMR (Supplementary Figures 4,5). Peak integrations (A), calibrated to the three protons of the terminal methyl group on 3-hydroxyoctanoate (3HO) monomers (δ 0.88), were used to determine the ratio of the DN_3_ protons in the alpha position (with respect to the azido group, δ 3.2, 2H) to the sum of the integrations of both chemical shifts (Sperling, 2015):

$$\chi_{D\,N_{3}}=\frac{\frac{A_{3.2}}{2}}{\frac{A_{3.2}}{2}+\frac{A_{0.88}}{3}}$$

The PHODN_3_ yield (g L^–1^) for trial 24 (Table 2) was calculated by first obtaining the yield of the 3HO fraction using GC as described above. This was multiplied by the ratio of the mole fractions of DN_3_ and 3HO to find the yield of the DN_3_ fraction, and added to the yield of 3HO:

$${\text{PHODN}}_{3}\,\text{yield}\,\left(g\,L^{-1}\right)=3\,\mathrm{HO}\,\left(g\,L^{-1}\right)\times \frac{\left(M\,W_{D\,N_{3}}\times \chi_{D\,N_{3}}\right)}{\left(M\,W_{3\,H\,O}\times \chi_{3\,H\,O}\right)}+3\,H\,O\,\left(g\,L^{-1}\right)$$

## Results

### Preliminary Trials and the Effect of pH

The goal of this investigation was to enhance the production of MCL PHA by developing high density fermentation methods for our engineered production platform, *E. coli* LSBJ. Previous attempts by this lab have been unsuccessful using LSBJ (unpublished data). Preliminary trials (trials 1–8, Table 2) sought to address this by generating several further mutations hypothesized to eliminate negative regulation of the PHA biosynthesis pathway. The mutant strains LSBJ CRP^∗^, SM02, SM04, and SM06 were developed and cultured by high-density fermentation in an attempt to produce either poly(3-hydroxyoctanoate), poly(3-hydroxydecanoate), or poly(lactate-*co-*3-hydroxyoctanoate; PHO, PHD, and PLA-co-PHO; Figure 4A). Despite some improvement in biomass accumulation, no observable polymer was produced in any of these experiments.

Based on previous observations that LSBJ would not produce PHA during bioreactor fermentation unless the pH was left uncontrolled, LSBJ was cultured in baffled shake flasks supplemented with decanoic acid using typical PHA biosynthesis conditions (Scheel et al., 2016), and both pH and PHA yield were measured every 12 h during growth (Figure 4B). The pH of culture media increased substantially over 48 h to a final pH of 8.8. PHA was present after 12 h at 6.01% of dry cell weight, and more than tripled to 19.7% by 24 h while the pH increased to 8.5. The amount of PHA continued to increase at a pH between 8.5 and 8.8 for the remainder of the experiment. These results suggest a correlation between an alkaline pH and PHA biosynthesis from fatty acids.

Based on our findings from the shake flask experiment, LSBJ harboring pBBRC1J4SII was cultured in the bioreactor and supplemented with decanoic acid, and upon initiating Stage 2 the pH was increased to 8.0 and maintained there for the duration of the fermentation. PHD was observed by GC analysis at a concentration of 0.16 g L^–1^, which was confirmed by NMR spectroscopy on purified polymers obtained by Soxhlet extraction due to the presence of characteristic chemical shifts at 5.15 and 2.5 ppm (Figure 4C).

### Optimization of PHD Production

**FIGURE 5:**
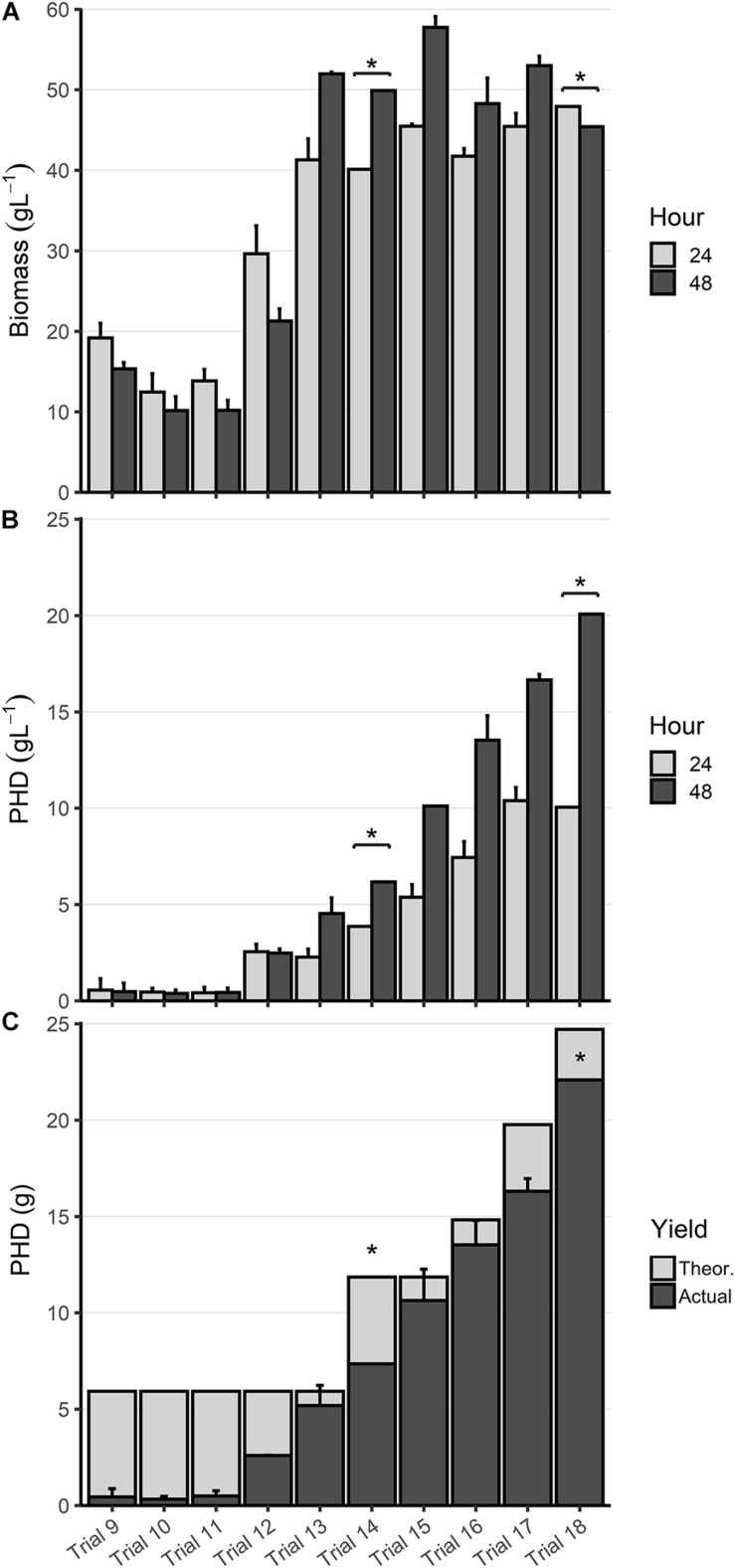
Optimization of PHD biosynthesis. **(A)** Biomass and **(B)** PHD accumulation (g L^1^) at 24 (light gray) and 48 h (dark gray) from bioreactor fermentations. **(C)** Total PHD yield in grams (dark gray), displayed as an overlay of the maximum theoretical yield (light gray). All data shown is the mean of duplicate trials, plus 1 standard deviation about that mean, except for two experiments where *n* = 1 (indicated by *). Trial numbers are labeled along the *x*-axis, which corresponds to the information in Table 2. Experiments were initially performed with 6 g of decanoic acid in the fatty acid feed, which was increased in later experiments as described in Table 2.

After successfully producing PHD in trial 9, mutant strains RSC02 (Δ*arcA*) and LSBJ CRP^∗^ were analyzed to determine the effects of removing these transcriptional regulators (Trials 10 and 11, Table 2). Little difference was observed between LSBJ (Trial 9), RSC02 (Trial 10), and LSBJ CRP^∗^ (Trial 11); 0.48, 0.39, and 0.44 g L^–1^ of PHD were produced by each strain after 48 h, respectively, with no observable difference between 24 and 48 h, and high variation between duplicate fermentations (Figure 5B). Dry biomass yield decreased for both mutants relative to LSBJ, reflecting a higher weight percent of PHD, and biomass decreased between 24 h and 48 h for all three strains (Figure 5A). Total polymer accumulation after 48 h was low relative to the maximum theoretical yield, ranging from 5.6 to 8.3% with high variation between duplicate fermentations (Figure 5C). Residual decanoic acid was present in the supernatant at a high level, ranging from 2.31 to 3.11 g with a large amount of variance (Table 3).

**TABLE 3:** PHD molecular weight and composition from optimization experiments.

Trial^1^	Residual decanoic	Molecular weight			3HD
	Acid (g)^2^	Mn (kDa)	Mw (kDa)	PDI (Mw/Mn)	(mol%)
9	2.310 ± 0.722	28.6 ± 14	81.5 ± 54	2.7 ± 0.6	100
10	3.101 ± 1.362	64.3 ± 25	157 ± 24	2.6 ± 0.6	100
11	3.113 ± 0.188	40.2 ± 6.4	127 ± 4.4	3.2 ± 0.4	100
12	1.578 ± 0.249	31.6 ± 9.8	80.4 ± 29	2.5 ± 0.1	100
13	0.079 ± 0.016	35.9 ± 4.8	81.0 ± 9.5	2.3 ± 0.1	100
14	0.023	39.8	106.1	2.7	100
15	0.036 ± 0.051	44.7 ± 4.7	125 ± 4.4	2.8 ± 0.4	100
16	0.022 ± 0.004	37.3 ± 0.1	117 ± 0.6	3.1 ± 0	100
17	0.027 ± 0.016	37.3 ± 4.8	103 ± 26	2.8 ± 0.4	100
18	2.332	39.3	133	3.4	100

*^1^All trials except 14 and 18 were performed in duplicate, and data is shown as the average ± the standard deviation of the two fermentations.*

*^2^Residual decanoic acid was measured in the supernatant after 48 h of growth.*

Next, we reduced the rate of glucose feed addition during Stage 2 to 9.7 mL h^–1^ and cultured LSBJ harboring pBBRC1J4SII to determine the effect on PHD biosynthesis (Trial 12, Figure 5). Biomass yield increased by approximately 50% at 24 h relative to Trial 9 (29.6 g L^–1^); biomass still decreased between 24 h and 48 h to a final concentration of 21.3 g L^–1^ (Figure 5A). PHD accumulation increased considerably to 2.48 g L^–1^ after 48 h, with no observable difference between 24 h and 48 h (Figure 5B). Total polymer accumulation after 48 h was 2.60 g, a yield of 43.8% (Figure 5C). Residual decanoic acid was reduced in the supernatant to 1.58 g (Table 3).

With bioreactor fermentations continuing to show a decrease of biomass and unchanged PHA accumulation between 24 and 48 h, believed to be due to culture death, the bioreactor medium was changed to that described in *high-density fed-batch production* to support greater growth of LSBJ harboring pBBRC1J4SII (Trial 13, Figure 5). Biomass yield increased relative to Trial 12 to 41.3 and 52.0 g L^–1^ at 24 and 48 h, respectively (Figure 5A). PHD accumulated at a concentration of 2.27 g L^–1^ by 24 h, which doubled to 4.54 g L^–1^ by 48 h (Figure 5B). Total polymer accumulation after 48 h was 5.12 g, a yield of 87.5% (Figure 5C). Little fatty acid remained in the supernatant after 48 h (0.079 g, Table 3).

With PHD yields approaching the theoretical maximum, we increased the amount of decanoic acid added to the feed solution from 6.0 to 12.0 g (Trial 14, Figure 5). This resulted in increased PHD yields of 3.87 and 6.18 g L^–1^ at 24 and 48 h, respectively, with a total yield of 7.35 g, or 62.0% of the theoretical maximum (Figures 5B,C). Biomass accumulation decreased slightly relative to Trial 13 in response to the additional fatty acid (Figure 5A). Residual decanoic acid remained low, with only 0.023 g detected (Table 3). To further support cell growth and PHA biosynthesis, the amount of yeast extract added to the culture medium was increased from 1.5 to 3.0 g (Trial 15, Figure 5). This again resulted in increased PHD yields, up to 5.38 and 10.1 g L^–1^ at 24 h and 48 h, bringing the total yield to 10.6 g (89.7%), with only 0.036 g of residual decanoic acid (Table 3). As the amount of decanoic acid was increased from 12 to 25 g over subsequent trials (Trials 16–18), PHD accumulation continued to increase as well until reaching 20.1 g L^–1^ at 48 h, a total yield of 22.1 g (89.4%), at which point excessive foaming became an issue and the optimization process was halted (Figures 5B,C). Measurements of decanoic acid remaining in the supernatant remained low for Trials 16 and 17 (0.022 and 0.027 g, respectively), and increased for Trial 18 (2.33 g, Table 3).

Purified polymers from each optimization experiment were analyzed by GPC to determine the number average molecular weight (M_*n*_), weight average molecular weight (M_*w*_), and polydispersity index (PDI, M_*w*_/M_*n*_; Table 3). M_*n*_ values ranged from 28.6 to 64.3 kDa with no discernable trend and a high degree of variation. The polydispersity index was similarly variable, ranging from 2.3 to 3.9.

### Production of Alternative MCL PHA Polymers

**FIGURE 6:**
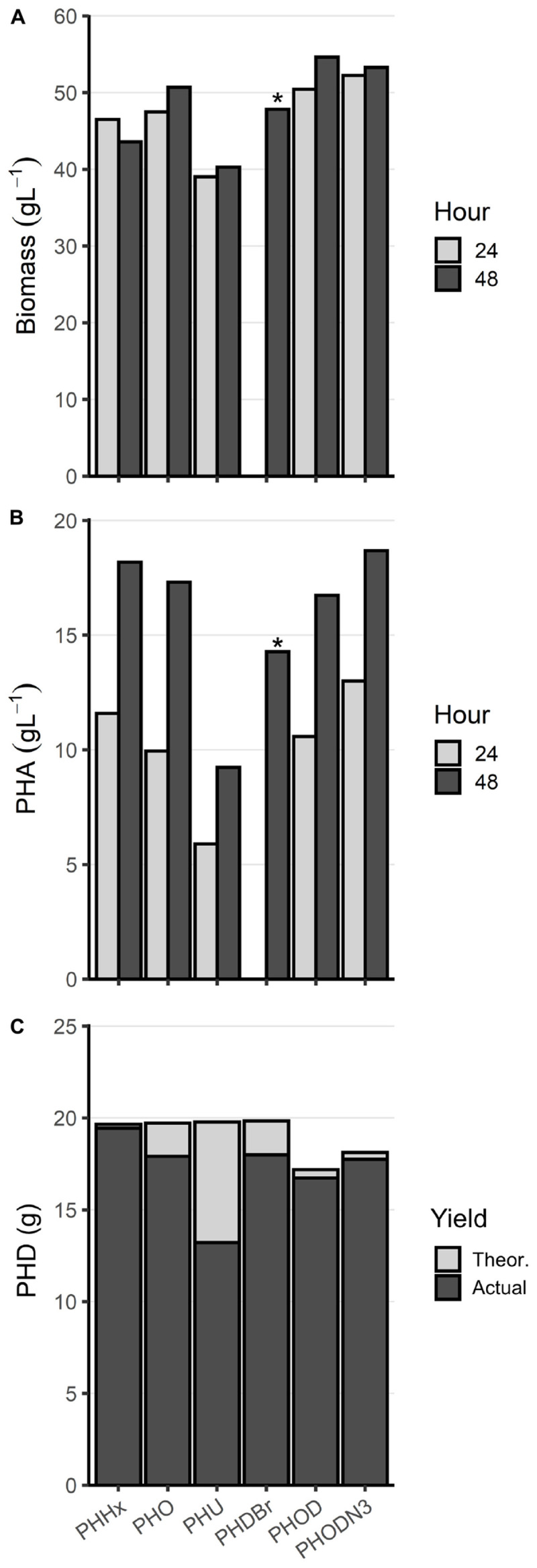
Alternative MCL PHA biosynthesis from Trials 19–24. **(A)** Biomass and **(B)** PHA accumulation (g L^1^) at 24 (light gray) and 48 h (dark gray) from bioreactor fermentations. **(C)** Total PHA yield in grams (dark gray), displayed as an overlay of the maximum theoretical yield (light gray). Trials were not performed with replicates and demonstrate proof-of-concept for expanding the possible substrates for the optimized high-density fermentation process. The asterisk (*) above PHDBr denotes missing data for the 24 h timepoint. Abbreviations: PHHx, poly(3-hydroxyhexanoate), Trial 19; PHO, poly(3-hydroxyoctanoate), Trial 20; PHU, poly(3-hydroxy-10-undecenoate, Trial 21; PHDBr, poly(3-hydroxy-10-bromodecanoate), Trial 22; PHOD, poly(3-hydroxyoctanoate-*co*-3-hydroxydecanoate), Trial 23; PHODN_3_, poly(3-hydroxyoctanoate-*co*-3-hydroxy-10-azidodecanoate), Trial 24.

To further investigate the capabilities of the optimized high-density fermentation process, LSBJ harboring pBBRC1J4SII was fed one of two alternative saturated MCL fatty acids in place of decanoic acid: hexanoic acid and octanoic acid (Trials 19 and 20, Table 2). Feed rates and fatty acid concentrations (20 g in 200 mL) were kept the same as Trial 17 to avoid the foaming issue observed at higher decanoic acid concentrations (Trial 18). High biomass accumulation was observed for both trials, similar to biomass yields obtained for Trial 17 (Figure 6A). Both fatty acids were successfully converted into their corresponding polymers, poly(3-hydroxyhexanoate) [PHHx] and poly(3-hydroxyoctanoate) [PHO], with yields of 18.2 and 17.3 g L^–1^ after 48 h, respectively, and followed a similar trend of increased PHA accumulation between 24 and 48 h (Figure 6B). Total polymer accumulation approached the maximum theoretical yield for both PHHx and PHO (99.0% and 90.8%; Figure 6C). The molecular weights of the resulting polymers were higher than those obtained for PHD, while the PDI remained consistent (Table 4).

**TABLE 4:** Alternative MCL PHA molecular weights and composition.

Trial	Polymer	Mn (kDa)	Mw (kDa)	PDI	Polymer composition (mol%)					
					3HX	3HO	3HD	3HU	3HDBr	3HDN_3_
19	PHHx	117.8	334.9	2.8	100	0	0	0	0	0
20	PHO	66.3	192.3	2.9	0	100	0	0	0	0
21	PHU	39.1	132.9	3.4	0	0	0	100	0	0
22	PHDBr	21.6	70.5	3.3	0	0	0	0	100	0
23	PHOD	104.7	286.7	2.7	0	81.8	18.2	0	0	0
24	PHODN_3_	47.1	149.0	3.1	0	80.4		0	0	19.6

Biosynthesis of functionalized PHAs, desirable materials that are more challenging to produce, was also attempted using the optimized high-density fermentation process (Trial 21 and 22, Table 2). Utilizing the same culture conditions and inoculum, 10-undecenoic acid and 10-bromodecanoic acid were supplemented to produce their corresponding polymers, poly(3-hydroxy-10-undecenoate; PHU) and poly(3-hydroxy-10-bromodecanoate; PHDBr; Figure 6). PHU yield was lower at 48 h compared to other polymers at 9.24 g L^–1^, although due to excessive foaming and subsequent antifoam addition the final culture volume was high and the total polymer accumulation was 13.2 g (66.8% of theoretical, Figures 6B,C). Similarly, 10-bromodecanoic acid caused excessive foaming and resulted in a lower yield and higher final volume, although to a lesser extent; PHDBr yield at 48 h was 14.3 g L^–1^ with a total accumulation of 18.0 g (90.7%). The M_*n*_ of PHU was similar to that of PHD at 39.1 kDa, while the M_*n*_ of PHDBr was low at 21.6 kDa (Table 4).

Biosynthesis of copolymers was also demonstrated using the high-density fermentation process by supplementing the feed with two fatty acids simultaneously. Octanoic acid was first mixed with decanoic acid (Trial 23), then with 10-azidodecanoic acid (Trial 24), both with 80 mol% octanoic acid (0.58 M total fatty acid). The copolymers poly(3-hydroxyoctanoate-*co*-3-hydroxydecanoate; PHOD) and poly(3-hydroxyoctanoate-*co*-3-hydroxy-10-azidodecanoate; PHODN_3_) were both produced successfully, with yields at 48 h of 16.7 and 18.7 g L^–1^ and total accumulation of 16.7 (97.4%) and 17.8 g (97.9%), respectively (Figures 6B,C). The M_*n*_ of PHOD was 105 kDa, and the M_*n*_ of PHODN_3_ was 47.1 kDa (Table 4).

## Discussion

The goal of this study was to develop a fed-batch high-density fermentation process capable of producing MCL PHA with controlled monomer compositions at a higher titer than that previously achieved in shake flasks. Based on the evidence in Figures 5, 6B, this process offers a significant improvement over previous shake flask studies, and a variety of MCL PHA homopolymers and copolymers were made successfully (Tappel et al., 2012b; Scheel et al., 2016).

In initial experiments with high-density fermentation of LSBJ, including those published here as well as unpublished data from our research group, we were unable to detect any evidence of PHA biosynthesis. Preliminary experiments sought to address this through a series of genetic engineering experiments, beginning with the creation of LSBJ CRP^∗^. In LSBJ, which has a wild-type CRP, catabolite repression plays an important role in regulating primary and secondary metabolism (Perrenoud and Sauer, 2005; Franchini et al., 2015). In the bioreactor fermentation, which used glucose as the carbon source, we hypothesized that low levels of CRP-cAMP were preventing the expression of β-oxidation genes necessary for PHA biosynthesis, and that conferring the CRP^∗^ mutation to LSBJ would alleviate this repression (Clark, 1981; Wade et al., 2001; Khankal et al., 2009). This was not found to be the case in initial experiments (data not shown), and further mutations were made to this strain in the subsequent preliminary experiments (Trials 1–8, Table 2, and Figure 4A).

Production of poly(lactate)-*co*-poly(hydroxyoctanoate) [PLA-*co*-PHO], a novel copolymer that has generated research interest, was attempted in Trial 1. Deletion of the *dld* gene from LSBJ CRP^∗^, which encodes for D-lactate dehydrogenase and has been shown to prevent the accumulation of PLA in *E. coli* (Taguchi et al., 2008), did not result in any detectable PLA-*co*-PHO. Although attempts to incorporate lactate monomers were abandoned in subsequent trials, the Δ*dld* mutant (SM02) was further engineered by deletion of the *arcA* gene. Deletion of *arcA* was previously shown to upregulate expression of β-oxidation genes and enhance MCL PHA biosynthesis, and we hypothesized that the removal of *arcA* combined with the CRP^∗^ mutation (SM04) would enable PHA biosynthesis during bioreactor fermentation (Scheel et al., 2016). Again, this was not supported by the evidence, with no detectable polymer observed (Trial 2).

We next hypothesized that the exogenous octanoic acid was being directed toward fatty acid biosynthesis rather than degradation, and that this could be prevented by deletion of the *aas* gene encoding for acyl-acyl carrier protein (acyl-ACP) synthetase. Acyl-ACP synthetase has a high affinity toward octanoate, and coupled with the increased membrane biosynthesis necessary for rapid culture growth during high-density fermentation it is possible that the octanoic acid was routed away from β-oxidation (Ray and Cronan, 1976; Black and DiRusso, 2003). However, the *aas*-deficient strain SM06 was unable to produce PHO from octanoic acid (Trial 3). Increasing the amount of octanoic acid available, both in bulk addition and continuous feeding strategies with SM06, likewise did not result in polymer accumulation (Trial 4–6). A stronger promoter, generated by converting the *lac* promoter of pBBRC1J4SII into the *tac* promoter by mutagenesis of the –35 region, was hypothesized to enable PHA biosynthesis by increasing expression of *phaJ4* and *phaC1* (Trial 7). This did not work, however, this is unsurprising as those two genes are under the control of a low-expressing constitutive *Cupriavidus necator* promoter inserted before the *lac* promoter of pBBRC1J4SII, and PHA biosynthesis has been demonstrated with this system numerous times during growth in shake flasks (Wang et al., 2012; Tappel et al., 2012b). Overexpression of the *atoDAEB* operon, which was previously shown to occur in the *arcA*-deficient LSBJ strain RSC02 (Scheel et al., 2016), was also unsuccessful in enabling PHA biosynthesis (Trial 8).

The effect of increasing the culture pH to 8.0 during Stage 2 is simultaneously the most profound and confounding result from this study (Trial 9). It was essential to the production of MCL PHA from exogenous fatty acids in LSBJ (Figure 4C); maintaining the pH at 7.0 prevented PHA accumulation in all preliminary trials (Figure 4A), as well as numerous trials not shown here. It is well-documented that growing *E. coli* to stationary phase in LB (and other tryptone-based media) commonly results in alkalinization, which explains the results of Figure 4B and could explain why shake flask production is successful at producing MCL PHA. The literature does not offer any clear connections between MCL fatty acid uptake and catabolism at an alkaline pH, although there is a paucity of research in this area relative to growth conditions at a neutral pH (Stancik et al., 2002; Yohannes et al., 2004; Maurer et al., 2005; Padan et al., 2005). Despite no clear connections, several aspects of *E. coli* pH homeostasis in alkaline environments may have an indirect effect on PHA biosynthesis. Numerous transport systems are up-regulated to direct the H^+^ flow into the cell, and FadD does transport H^+^ concomitantly with fatty acids (Weimar et al., 2002). No evidence of differential expression for *fadD* has been observed, however, transcriptome studies cannot capture proteins that are regulated at the activity level rather than expression, which is common for many Na^+^/H^+^ antiporters (Maurer et al., 2005; Padan et al., 2005). Another contributing factor could be the repression of the flagellar biosynthesis and chemotaxis regulons, which are down-regulated extensively (47 out of 50 genes, pH 8.7) at an alkaline pH (Maurer et al., 2005). Repression of this energy-intensive process could have freed up cellular resources such as ATP for PHA biosynthesis. Further research into fatty acid uptake and metabolism would be necessary to determine the direct effects of pH on PHA biosynthesis, which falls far beyond the scope of this study.

Although successful, the PHD yield in Trial 9 did not offer an improvement over typical shake flask production. To test the earlier hypotheses of low CRP-cAMP inhibiting biosynthesis and repression by ArcA, we repeated the conditions used in Trial 9 with both RSC02 (Trial 10) and LSBJ CRP^∗^ (Trial 11). No benefit to PHD yield was observed for either Trial; PHD weight percent was improved slightly, but this was offset by a reduced biomass (Figures 5A,B). For each of these trials free fatty acid remained in the media, though not in high enough amounts to account for the yield of PHA observed (Table 3). This suggests that fatty acid uptake is not the limiting factor, but rather the conversion of internalized fatty acids and their intermediates into PHA.

Reducing the rate of glucose addition during Stage 2 (Trial 12) increased the yield of PHD to 2.48 g L^–1^, greater than a 5-fold improvement (Figure 5B). One explanation for this is the overabundance of glucose in previous trials and the subsequent increase in overflow metabolism. Overflow metabolism in *E. coli* occurs during high levels of aerobic glucose consumption, and results in the excretion of acetate which can become toxic and growth-limiting (Vemuri et al., 2006). This is particularly pronounced in *E. coli* K-12 strains, which LSBJ is derived from, due to an inactive or low-activity glyoxylate shunt (van de Walle and Shiloach, 1998; Noronha et al., 2000; Rand et al., 2017). An increase in biomass of approximately 50% provides additional evidence to support this explanation, however, this biomass increase is not enough to account for the magnitude of the increase in PHD yield (Figure 5A). Another effect of the reduced glucose feed rate could be an increase in the cAMP pool resulting from increased adenylate cyclase activity due to decreased glucose transport (Notley-McRobb et al., 1997). This in turn would increase CRP-cAMP abundance and could lead to upregulation of *fadD* and *fadE*, resulting in enhanced PHD biosynthesis.

One issue that needed to be addressed was the apparent death of the bacterial culture during Stage 2, as evidenced by a reduction in biomass and lack of PHA accumulation between 24 and 48 h (Figures 5A,B), and by a marked decrease in oxygen consumption as early as 13 h until DO% remained nearly saturated (Supplementary Figures 6–10). Previous research has indicated that high ammonia concentrations during high-density fermentation inhibit the growth of *E. coli* and limit secondary metabolism, which could explain the results observed in this study (Riesenberg et al., 1991; Lau et al., 2004; Shiloach and Fass, 2005). Reformulating the culture medium and glucose feed based on low ammonia alternatives described by Lau et al. (2004) appeared to eliminate the issue of culture death (Trial 13); biomass and PHD yield increased between 24 and 48 h (Figures 5A,B), oxygen demand slowly increased over the entire fermentation (Supplementary Figure 11), and total PHD accumulation at 48 h reached 87.5% of the theoretical yield (Figure 5C). Fatty acid consumption increased dramatically as well, with only 0.079 g of decanoic acid remaining in the media (Table 3). In addition to drastically reducing the ammonium concentration, the new formulation contained more phosphate to support a higher cell density and more trace metals to ensure adequate cofactor abundance (Herbert et al., 1965; Neidhardt et al., 1974).

With a media formulation capable of supporting high-density fermentation and the majority of fatty acid substrate being converted to polymer, the next logical step was the optimization of the fatty acid feed; specifically, increasing the amount and rate of decanoic acid addition (Trials 14–18). Progressively increasing the amount of decanoic acid added to totals of 12, 15, 20, and 25 g (addition rates of 0.44, 0.55, 0.65, and 0.75 g h^–1^, respectively) correlated with an increase in PHD yield, culminating in a final titer of 20.1 g L^–1^ (Figure 5B). However, adding 25 g of decanoic acid resulted in excessive foaming likely as a result of exogenous decanoate behaving as a surfactant, and replication of this feeding strategy proved to be difficult due to uncontrollable foaming and loss of culture. Alternative strategies exist to control foam in an industrial bioprocess setting, such as mechanical disruption or ultrasound, but these are incompatible with the small benchtop vessel used in this study (Delvigne and Lecomte, 2009; Routledge, 2012). As such the optimization of the fatty acid feed was halted, and analysis of previous metabolic engineering strategies (CRP^∗^, Δ*arcA*, etc.) was deemed unnecessary due to the physical nature of the foaming limitation. It is also important to note that the amount of yeast extract added to the initial culture medium was doubled to 3.0 g for Trial 15 and for each subsequent Trial. This was in response to a decrease in % yield when decanoic acid was increased to 12 *g*, believed to be due to an increased fatty acid toxicity. Fadzil et al. has previously shown that increasing the concentration of yeast extract for *E. coli* LSBJ cultures utilizing co-carbon sources (glucose and decanoic acid) increases their tolerance toward fatty acids and enhances MCL PHA biosynthesis (2018). Our results support this observation; increasing the concentration of yeast extract led to improved biomass, PHD yield, and decanoic acid utilization (89.7% yield; Trial 15, Figure 5).

The optimized high-density fermentation process developed here was also shown to be flexible with regard to fatty acid substrate; hexanoic acid, octanoic acid, 10-undecenoic acid, 10-bromodecanoic acid, and 10-azidodecanoic acid were all successfully polymerized in addition to decanoic acid (Figure 6), with confirmation by NMR for the functionalized PHU, PHDBr, and PHODN_3_ (Supplementary Figures 2–5). Each alternative MCL PHA tested was produced at yields of 9.24 g L^–1^ or higher and with no detectable co-monomers present except for the intentional copolymers PHOD and PHODN_3_. Interestingly, while PHU yields were substantially lower than the other MCL polymers there was very little 10-undecenoic acid remaining in the media after 48 h (0.013 g, data not shown). This suggests that the fatty acid was taken up by LSBJ, but not fully converted to PHU, which could be indicative of reduced enzymatic activity toward this uncommon substrate. The two copolymers contained monomer compositions closely resembling the mol% of the feed solution (81.8 and 80.4% 3HO, respectively), which is unsurprising given the high fatty acid utilization observed (approximately 97% for both; Figure 6C). This control over monomer composition is one of the primary benefits to using *E. coli* LSBJ as the production host, particularly over a wide range of fatty acid substrates (Tappel et al., 2012b). Although numerous high-density cultivation methods have been reported using native producers such as *Pseudomonas* species, only PHD has previously been produced at near-homopolymeric compositions and high titers, and there are no reported bioprocesses for the production of PHHx, PHO, PHU, or PHDBr homopolymers to the best of our knowledge (Sun et al., 2007; Jiang et al., 2013; Follonier et al., 2015; Gao et al., 2018; Oliveira et al., 2020).

The molecular weights varied for the PHAs produced, and the PDIs ranged from 2.3 to 3.9 (Tables 3, 4). The molecular weights (M_*n*_) of PHD showed considerable variation, ranging between 28.6 and 64.3 kDa. This is comparable and slightly less than previous work performed in shake flasks, where M_*n*_ values of 61.5 to 111 kDa were observed (Tappel et al., 2012b; Mohd Fadzil et al., 2018). Similarly, the M_*n*_ value of PHU was slightly less than previously observed (39.1 vs. 60 kDa; Tappel et al., 2012a), while the M_*n*_ of PHHx was higher than previous reports (118 vs. 77 kDa; Scheel et al., 2016). Overall molecular weights are comparable to those from similar studies, however, with the high degree of variation and lack of replicates for alternative MCL PHAs only limited comparisons can be made.

The high-density fermentation process described here was developed to allow for MCL PHA biosynthesis with controlled monomer composition at a higher yield than shake flask production can accommodate. The productivity shown here of PHD biosynthesis from 25 g decanoic acid (0.42 g L^–1^ h^–1^) rivals that of the highest published productivity for nearly homopolymeric PHD (0.41 g L^–1^ h^–1^), although this previous work had issues with reproducibility (Gao et al., 2018). With *E. coli* LSBJ, reproducible, high MCL PHA yields were demonstrated from a variety of fatty acid substrates, both saturated and functionalized, showing that this is a versatile process capable of producing unique and valuable materials. If the physical limitation of excessive foaming can be overcome this process could be further enhanced in terms of productivity and yield, and further efforts with metabolic engineering may be useful.

## Data Availability Statement

The original contributions presented in the study are included in the article/Supplementary Material, further inquiries can be directed to the corresponding author/s.

## Author Contributions

RS, BL, and CN were responsible for the original research ideas and planning experiments. RS wrote the manuscript, with support by CN. RS conducted the majority of the experiments and analyses in this work, and supervised the contributions of TH, YK, JM, and SM. SM conducted the preliminary experiments in the bioreactor with support from BL. JM assisted with cell harvest and polymer extraction/purification with supervision by RS. YK assisted with functionalized polymer production experiments with supervision by RS. TH assisted with bioreactor preparation and assembly, cell growth monitoring, cell harvest, and polymer extraction/purification with direction from RS. All authors contributed to manuscript revision and read and approved of the submitted version.

## Conflict of Interest

The authors declare that the research was conducted in the absence of any commercial or financial relationships that could be construed as a potential conflict of interest.
