# Supplementary material for: Optimizing a Fed-Batch High-Density Fermentation Process for Medium Chain-Length Poly(3-Hydroxyalkanoates) in Escherichia coli
Source: Front Bioeng Biotechnol. 2021 Feb 26;9:618259. doi: 10.3389/fbioe.2021.618259 (PMC7953831; doi:10.3389/fbioe.2021.618259)
Supplement: Supplementary file 1 [file Data_Sheet_1.PDF]

Supplementary Material

1 NMR Spectra

The data presented here are from nuclear magnetic resonance (NMR) experiments performed to characterize polyhydroxyalkanoates (PHAs) and the synthesis of 10-azidodecanoic acid. ^{1}H NMR spectra were recorded on either a Bruker AVANCE III 600 MHz instrument or a Bruker AVANCE III HD 800 MHz instrument, and were calibrated using residual undeuterated solvents as internal reference (chloroform, δ = 7.26 ppm). Chemical shifts (δ) are reported in parts per million (ppm); NMR peak multiplicities are denoted by the following abbreviations: s = singlet, d = doublet, t = triplet, q = quartet, p = pentet, sext = sextet, dd = doublet of doublets, dt = doublet of triplets, m = multiplet, br = broad. Spectra were processed with Bruker TopSpin v3.5pI2.

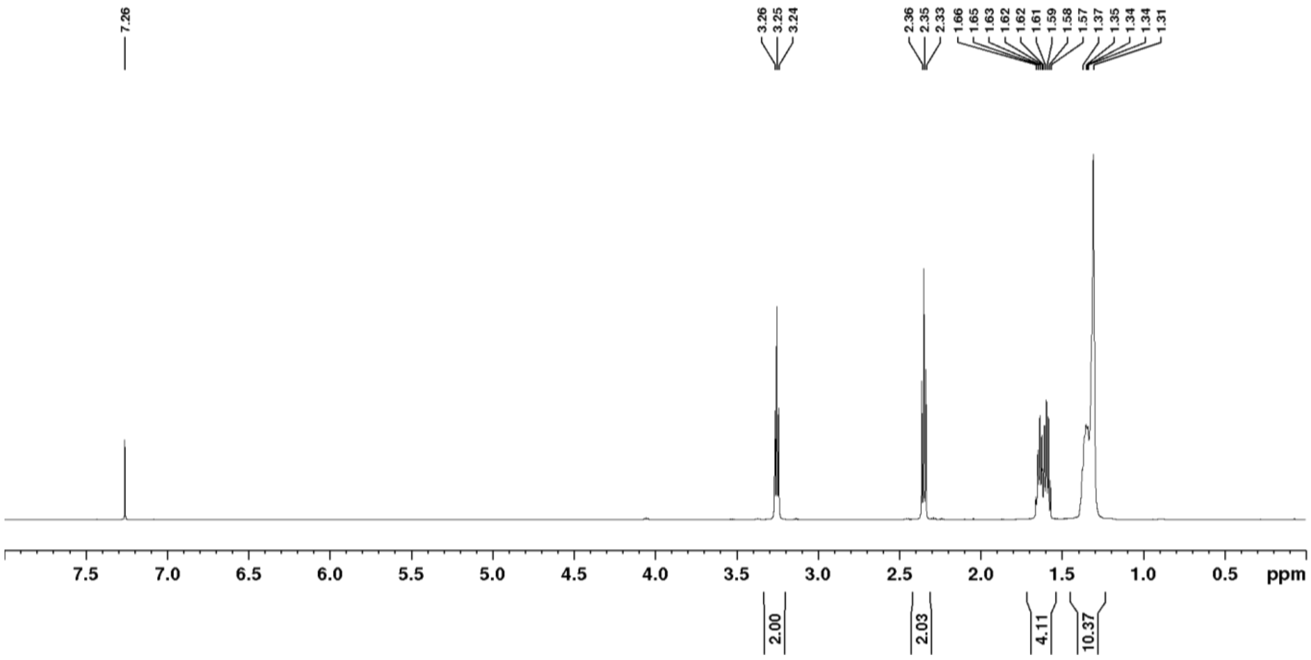

Supplementary Figure 1: ^{1}H-NMR (600 MHz, $CDCl_3$) spectrum of purified 10-azidodecanoic acid; δ 10.33 (br s, 1H, COOH), 3.27 (t, *J* 6.96 Hz, 2H, CH_2N_3), 2.38 (t, *J* 7.51Hz, 2H, CH_2COOH), 1.60-1.69 (m, 4H, CH_2), 1.30-1.41 (m, 10H, CH_2).

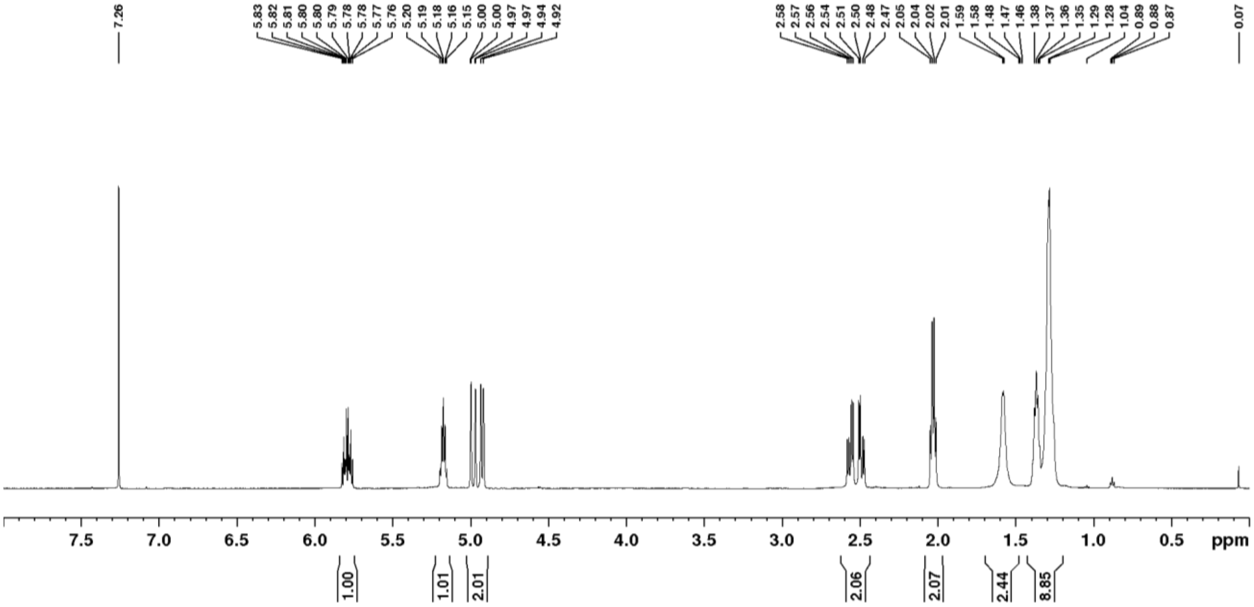

Supplementary Figure 2: ^{1}H-NMR (600 MHz, $CDCl_3$) spectrum of poly(*R*-3-hydroxy-10-undecenoate) extracted from *E. coli* LSBJ after bioreactor fermentation; δ 5.83-5.76 (m, 1H), 5.21-5.18 (p, 1H), 5.00-4.92 (dd, 2H), 2.59-2.48 (m, 2H), 2.05-2.01 (q, 2H), 1.60-1.54 (m, 2H), 1.38-1.28 (m, 8H).

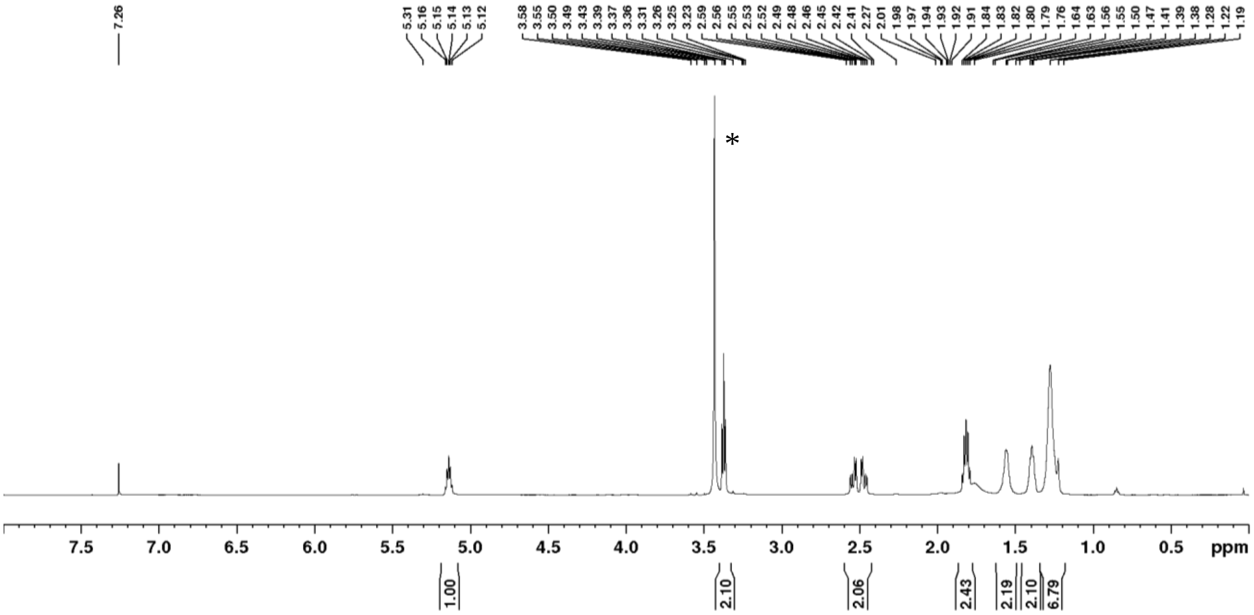

Supplementary Figure 3: ^{1}H-NMR (600 MHz, $CDCl_3$) spectrum of poly(*R*-3-hydroxy-10-bromodecanoate) extracted from *E. coli* LSBJ after bioreactor fermentation; δ 5.21-5.18 (p, 1H), 3.37 (t, 2H), 2.59-2.48 (m, 2H), 1.84-1.82 (p, 2H), 1.60-1.54 (m, 2H), 1.35-1.27 (m, 2H), 1.28 (m, 6H). The asterisk (*) at δ 3.43 denotes a methanol impurity (Fulmer et al., 2010).

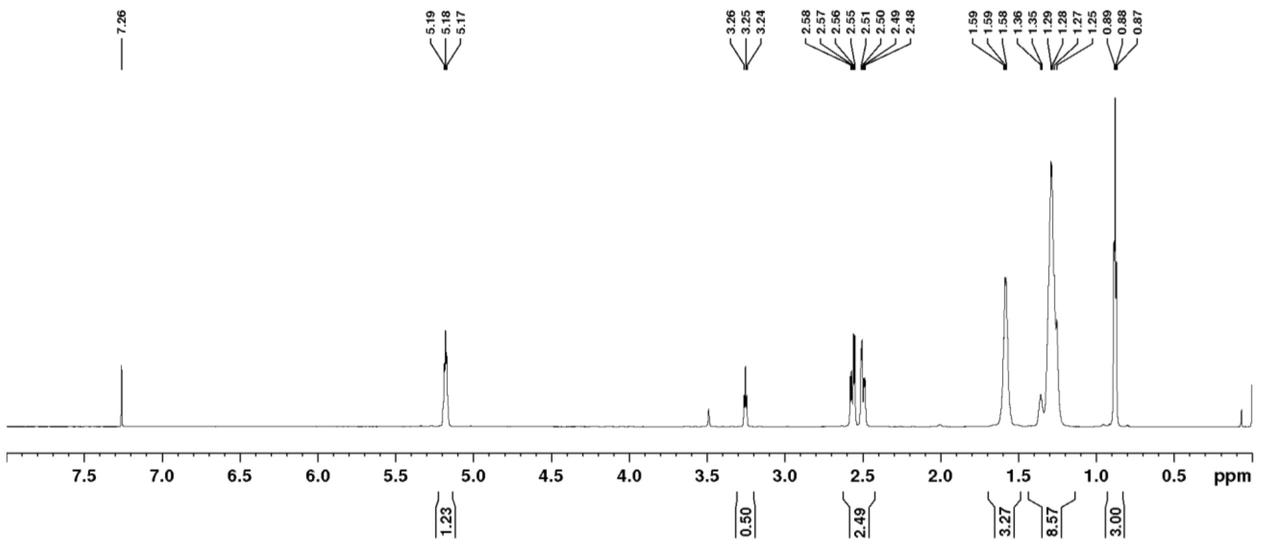

Supplementary Figure 4: ^{1}H-NMR (800 MHz, $CDCl_3$) spectrum of poly(*R*-3-hydroxyoctanoate-*co*-3-hydroxy-10-azidodecanoate) extracted from *E. coli* LSBJ after 24 h bioreactor fermentation; δ 5.19-5.17 (p, 1H), 3.26-3.24 (t, 1H), 2.58-2.48 (m, 2H), 1.59-1.58 (m, 2H), 1.36-1.25 (m, 6H/8H), 0.89-0.87 (t, 3H).

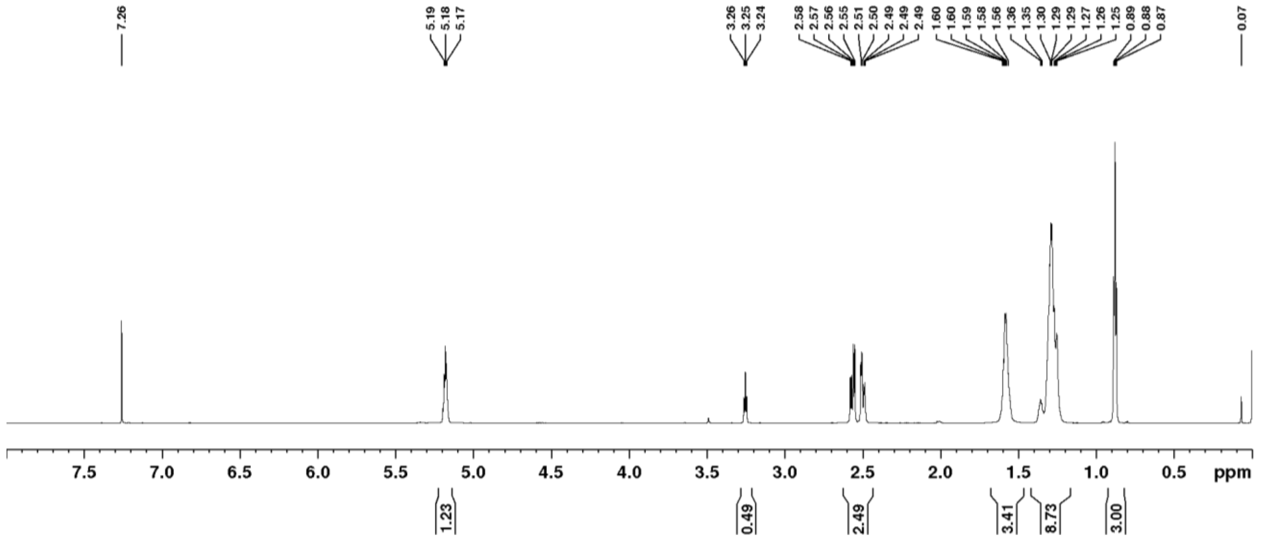

Supplementary Figure 5: ^{1}H-NMR (800 MHz, $CDCl_3$) spectrum of poly(*R*-3-hydroxyoctanoate-*co*-3-hydroxy-10-azidodecanoate) extracted from *E. coli* LSBJ after 48 h bioreactor fermentation; δ 5.19-5.17 (p, 1H), 3.26-3.24 (t, 1H), 2.58-2.48 (m, 2H), 1.59-1.58 (m, 2H), 1.36-1.25 (m, 6H/8H), 0.89-0.87 (t, 3H).

2 Bioreactor Traces

The representative data presented here are from the dissolved oxygen (DO), pH, temperature (T), agitation speed, and air sparging rate which were monitored and recorded every 30 seconds during each fermentation.

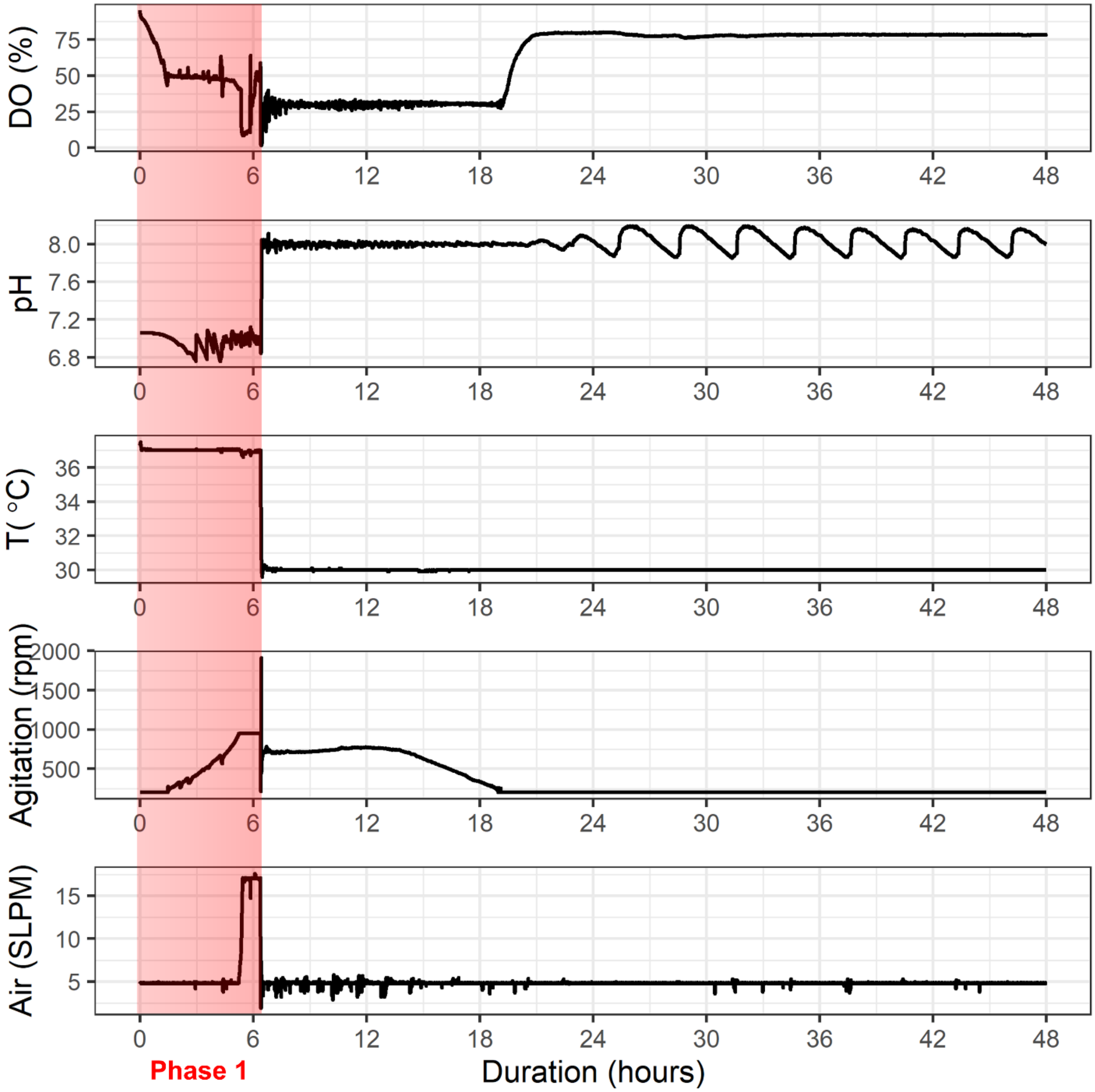

Supplementary Figure 6 (Trial 9A). Bioreactor fermentation of *E. coli* LSBJ harboring pBBRC1J4SII, with 6 g decanoic acid added at 0.22 g h^{-1}. Phase 1 is indicated by a red box, after which the culture was shifted to Phase 2 (OD_{600} of >30). Culture appeared to transition to the death phase after ~14-19 hours, evidenced by the decreased oxygen demand.

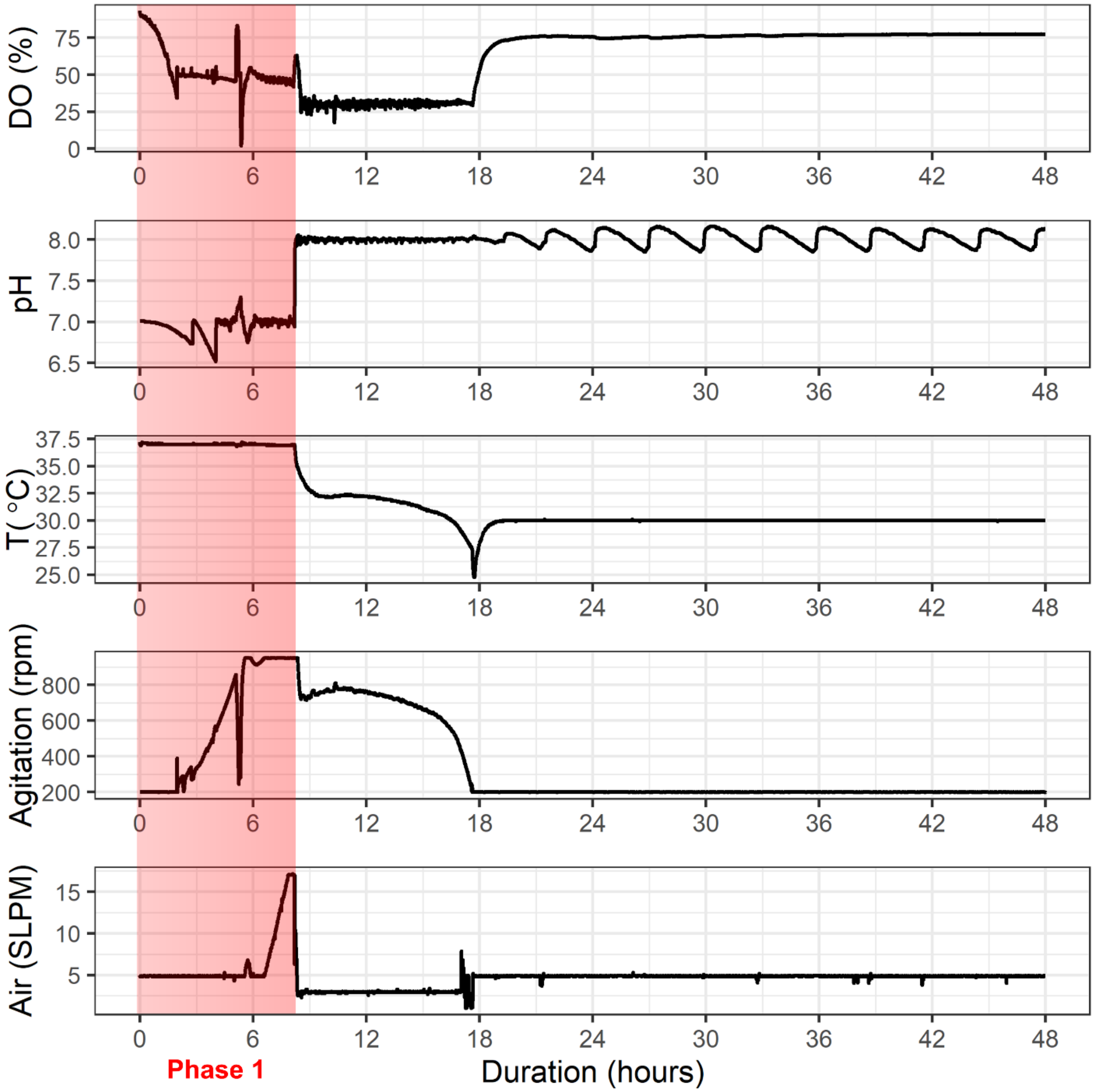

Supplementary Figure 7 (Trial 9B): Bioreactor fermentation of *E. coli* LSBJ harboring pBBRC1J4SII, with 6 g decanoic acid added at 0.22 g h^{-1}. The cooling system of the bioreactor had difficulty with the sudden decrease to 30 °C.

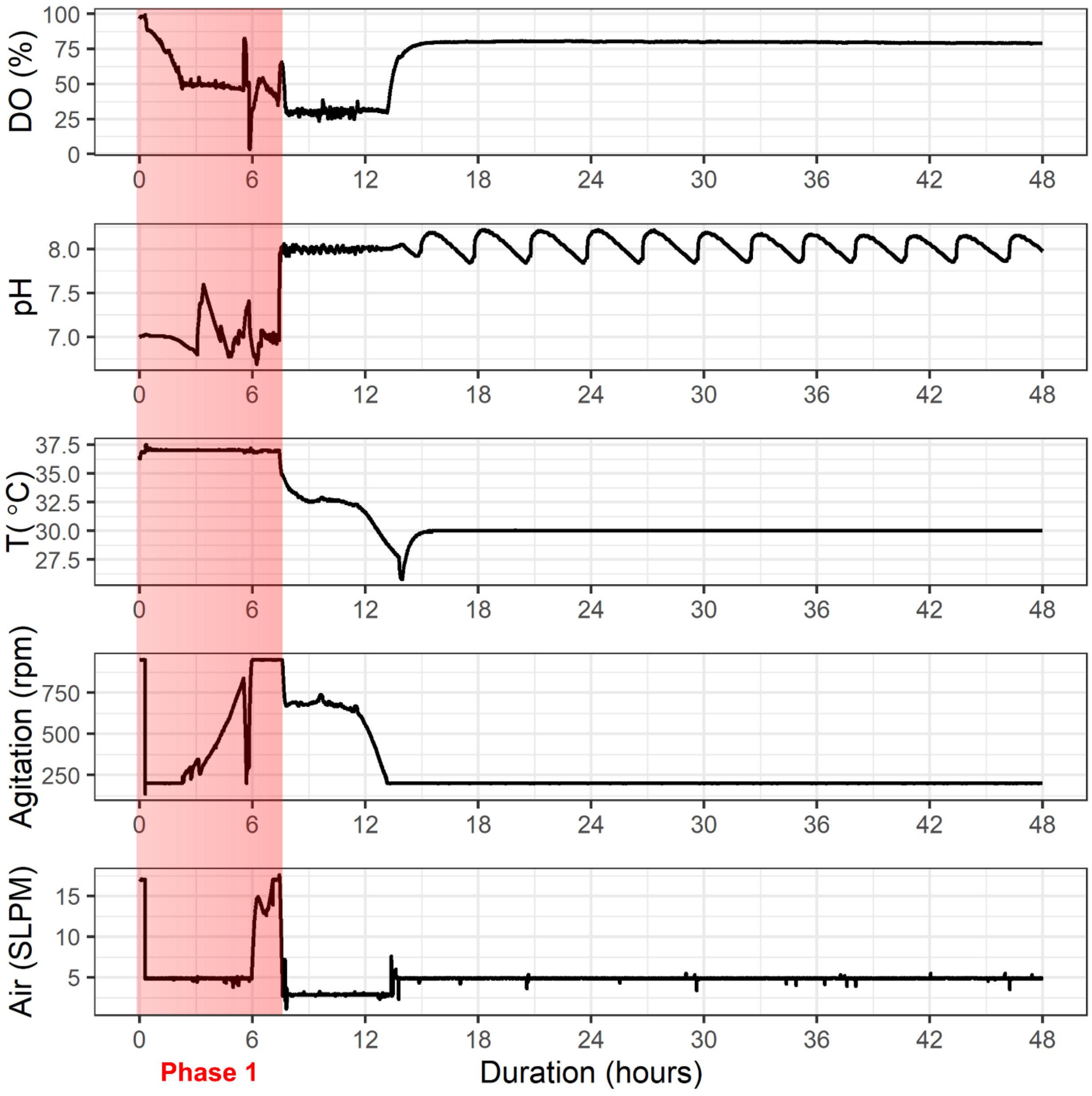

Supplementary Figure 8 (Trial 10A): Bioreactor fermentation of *E. coli* RSC02 harboring pBBRC1J4SII, with 6 g decanoic acid added at 0.22 g h^{-1}.

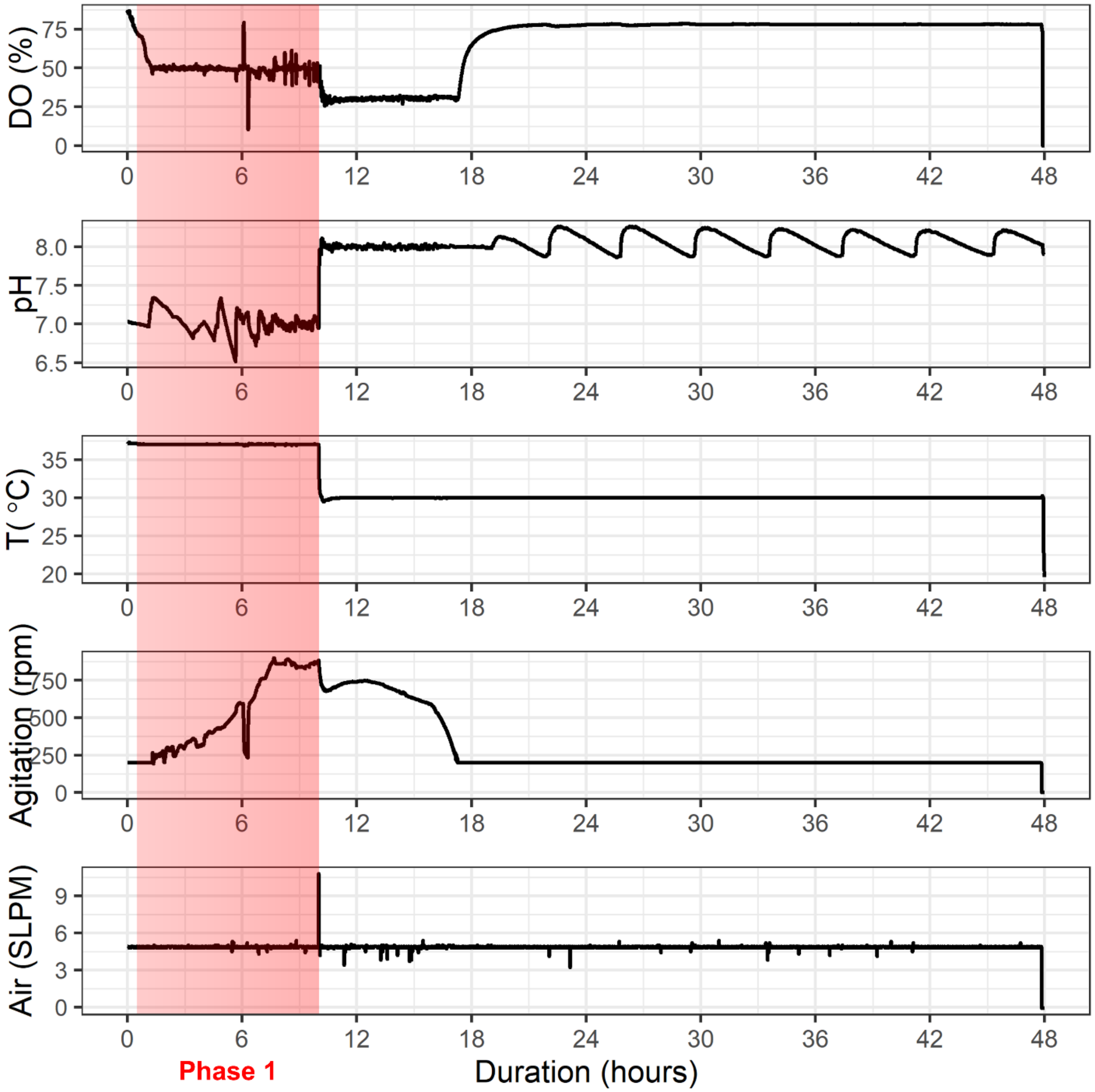

Supplementary Figure 9 (Trial 11A): Bioreactor fermentation of *E. coli* LSBJ CRP* harboring pBBRC1J4SII, with 6 g decanoic acid added at 0.22 g h^{-1}. Cold water input for the heat exchanger was adjusted to a lower temperature to better control the temperature at 30 °C.

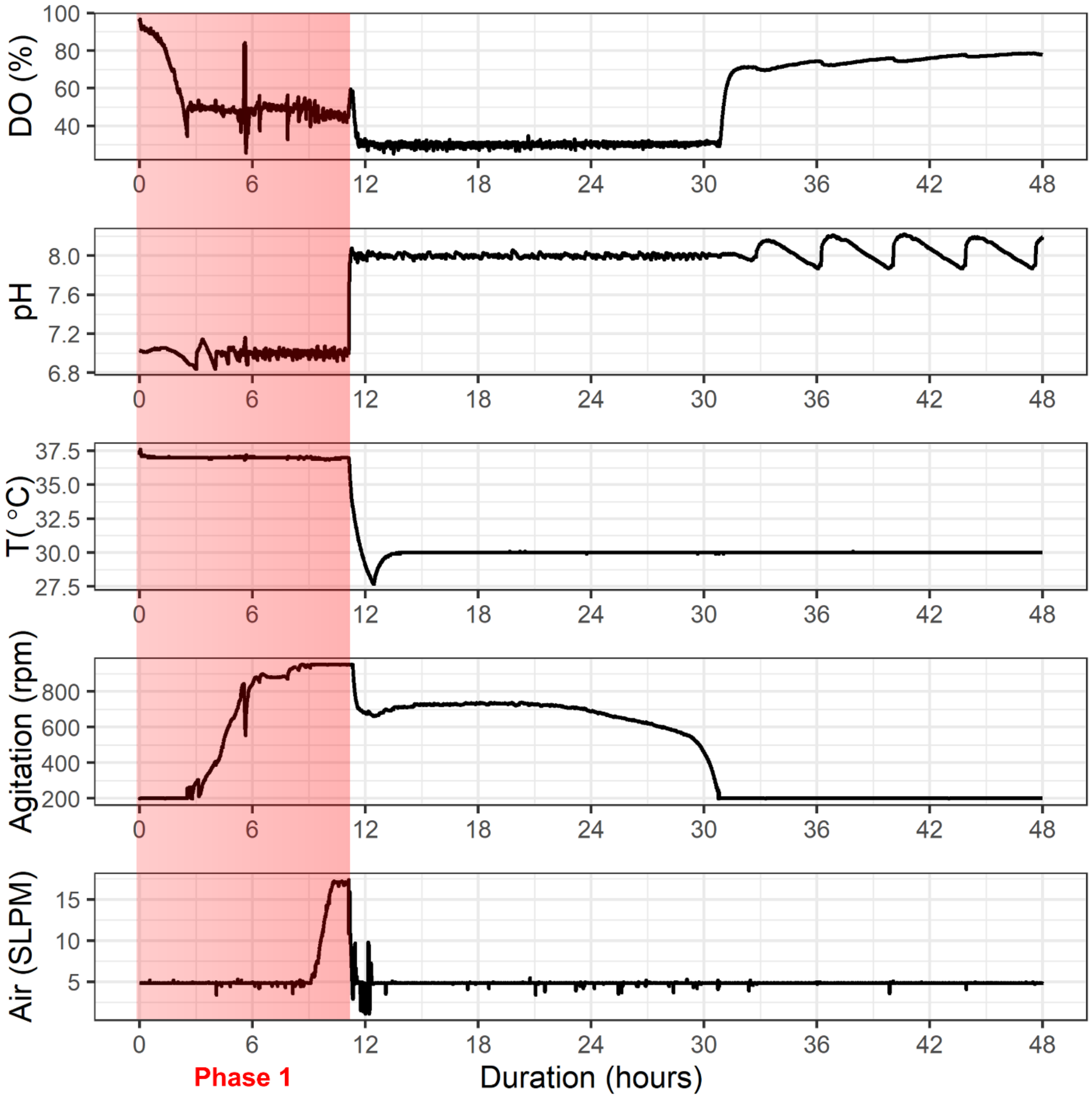

Supplementary Figure 10 (Trial 12A): Bioreactor fermentation of *E. coli* LSBJ harboring pBBRC1J4SII, with 6 g decanoic acid added at 0.22 g h^{-1}. Rate of glucose feed slowed to 9.7 mL h^{-1} during Stage 2, resulting in an increased culture growth time before its apparent death (low/zero oxygen consumption).

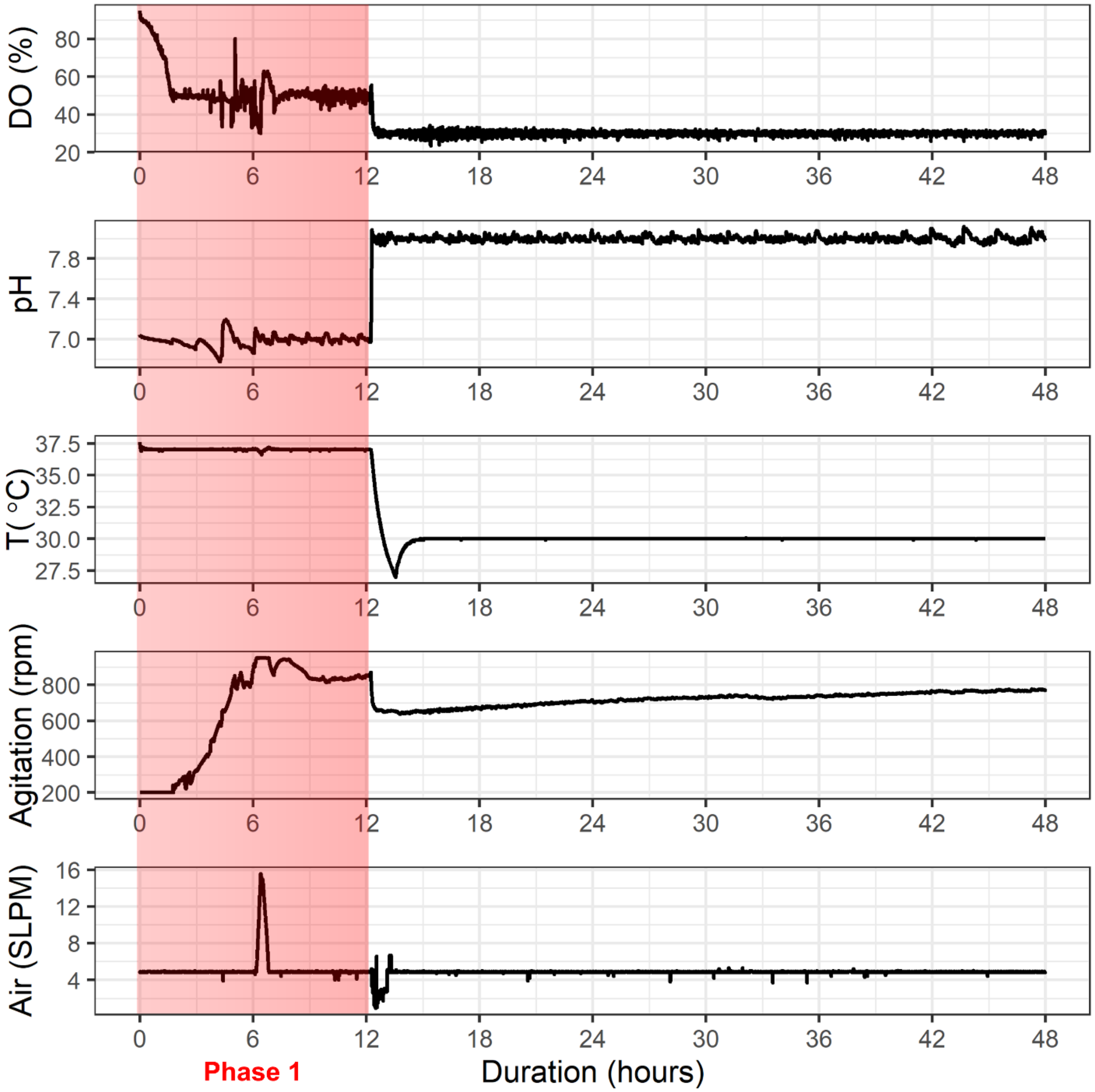

Supplementary Figure 11 (Trial 13A): Bioreactor fermentation of *E. coli* LSBJ harboring pBBRC1J4SII, with 6 g decanoic acid added at 0.22 g h^{-1}. Media formulation changed to that described in ***high-density fed-batch production***. Rate of glucose feed slowed to 9.7 mL h^{-1} during Stage 2. First run where culture survived and grew for the entire 48 h (O_2 demand slowly increased between 13 and 48 h, evidenced by the steadily increasing agitation rate needed to maintain DO at 30%).

3 References

Fulmer, G.R., Miller, A.J.M., Sherden, N.H., Gottlieb, H.E., Nudelman, A., Stoltz, B.M., Bercaw, J.E., Goldberg, K.I., 2010. NMR Chemical Shifts of Trace Impurities: Common Laboratory Solvents, Organics, and Gases in Deuterated Solvents Relevant to the Organometallic Chemist. Organometallics 29, 2176–2179. https://doi.org/10.1021/om100106e
